# Repair of acute respiratory distress syndrome by stromal cell administration (REALIST) trial: A phase 1 trial

**DOI:** 10.1016/j.eclinm.2021.101167

**Published:** 2021-10-24

**Authors:** Ellen Gorman, Manu Shankar-Hari, Phil Hopkins, William S. Tunnicliffe, Gavin D. Perkins, Jonathan Silversides, Peter McGuigan, Anna Krasnodembskaya, Colette Jackson, Roisin Boyle, Jamie McFerran, Cliona McDowell, Christina Campbell, Margaret McFarland, Jon Smythe, Jacqui Thompson, Barry Williams, Gerard Curley, John G. Laffey, Mike Clarke, Daniel F. McAuley, Cecilia M. O'Kane

**Affiliations:** a Wellcome-Wolfson Institute for Experimental Medicine, Queen's University Belfast, 97 Lisburn Road, Belfast BT9 7BL, United Kingdom; b Guy's and St Thomas’ NHS Foundation Trust, Westminister Bridge Road, London SE1 7EH, United Kingdom; c School of Immunology and Microbial Sciences, King's College London, Strand, London WC2R 2LS, United Kingdom; d Kings Trauma Centre, King's College London, Strand, London WC2R 2LS, United Kingdom; e Queen Elizabeth Hospital Birmingham, Mindelsohn Way, Edgbaston, Birmingham B15 2GW, United Kingdom; f Warwick Medical School, University of Warwick, Coventry CV4 7AL, United Kingdom; g University Hospitals Birmingham, Mindelsohn Way, Edgbaston, Birmingham B15 2GW, United Kingdom; h Belfast Health and Social Care Trust, Royal Victoria Hospital, 274 Grosvenor Road, Belfast BT12 6BA, United Kingdom; i Northern Ireland Clinical Trials Unit, 7 Lennoxvale, Belfast BT9 5BY, United Kingdom; j NHS Blood and Transplant, Headley Way, Oxford OX3 9BU, United Kingdom; k NHS Blood and Transplant Service, Vincent Drive, Edgbaston, Birmingham B15 2SG, United Kingdom; l Independent Patient and Public Representative, United Kingdom; m Royal College of Surgeons in Ireland, Dublin 9, Ireland; n Regenerative Medicine Institute (REMEDI) at CÚRAM Centre for Research in Medical Devices, National University of Ireland Galway, University Road, Galway H91 TK33, Ireland; o Northern Ireland Methodology Hub, School of Medicine, Dentistry and Biomedical Sciences, Queen's University Belfast, 97 Lisburn Road, Belfast BT9 7BL, United Kingdom

## Abstract

**Background:**

Mesenchymal stromal cells (MSCs) may be of benefit in acute respiratory distress syndrome (ARDS) due to immunomodulatory, reparative, and antimicrobial actions. ORBCEL-C is a population of CD362 enriched umbilical cord-derived MSCs. The REALIST phase 1 trial investigated the safety and feasibility of ORBCEL-C in patients with moderate to severe ARDS.

**Methods:**

REALIST phase 1 was an open label, dose escalation trial in which cohorts of mechanically ventilated patients with moderate to severe ARDS received increasing doses (100, 200 or 400 × 10^6^ cells) of a single intravenous infusion of ORBCEL-C in a 3 + 3 design. The primary safety outcome was the incidence of serious adverse events. Dose limiting toxicity was defined as a serious adverse reaction within seven days. Trial registration clinicaltrials.gov NCT03042143.

**Findings:**

Nine patients were recruited between the 7th January 2019 and 14th January 2020. Study drug administration was well tolerated and no dose limiting toxicity was reported in any of the three cohorts. Eight adverse events were reported for four patients. Pyrexia within 24 h of study drug administration was reported in two patients as pre-specified adverse events. A further two adverse events (non-sustained ventricular tachycardia and deranged liver enzymes), were reported as adverse reactions. Four serious adverse events were reported (colonic perforation, gastric perforation, bradycardia and myocarditis) but none were deemed related to administration of ORBCEL-C. At day 28 no patients had died in cohort one (100 × 10^6^), three patients had died in cohort two (200 × 10^6^) and one patient had died in cohort three (400 × 10^6^). Overall day 28 mortality was 44% (*n* = 4/9).

**Interpretation:**

A single intravenous infusion of ORBCEL-C was well tolerated in patients with moderate to severe ARDS. No dose limiting toxicity was reported up to 400 × 10^6^ cells.

**Research in context**

**Evidence before this study**

Several phase 1 trials have reported traditionally manufactured plastic adherent Mesenchymal Stromal Cells (MSCs) are safe and well tolerated in patients with Acute Respiratory Distress Syndrome (ARDS). The phase 2 START (Stromal cells for ARDS Treatment) trial investigated bone marrow derived plastic adherent MSCs in ARDS and did not demonstrate efficacy. Several clinical trials of MSCs in ARDS, and COVID-19 ARDS, are ongoing. ORBCEL-C is a defined population of CD362-enriched umbilical cord-derived MSCs manufactured by advanced techniques. They have not been investigated in clinical trials of patients with ARDS previously.

**Added value of this study**

The REALIST (Repair of Acute Respiratory Distress with Stromal Cell Administration) phase 1 trial aimed to investigate the safety and tolerability of a single intravenous infusion of ORBCEL-C in patients with moderate to severe ARDS, prior to proceeding to a larger phase 2 trial evaluating efficacy. No dose limiting toxicity was observed in any dose cohort (100, 200, and 400 × 10^6^ cells), and a dose of 400 × 10^6^ cells was determined to be the maximum tolerated dose for the phase 2 study.

**Implications of all the available evidence**

Following completion of this phase 1 study, having demonstrated the safety of ORBCEL-C and determined the maximum tolerated dose, investigators have progressed to the REALIST phase 2 study, investigating a single intravenous infusion of 400 × 10^6^ ORBCEL-C in patients with moderate to severe ARDS. In light of the COVID-19 pandemic, an additional cohort of patients with ARDS due to COVID-19 will be recruited to the phase 2 study.

Alt-text: Unlabelled box

## 1 Introduction

Acute respiratory distress syndrome (ARDS) is characterised by hypoxaemia and bilateral radiographic opacities [1]. The mortality burden is high, between 35 and 45%, and there is considerable physical and psychological morbidity in survivors [2], [3], [4], [5]. ARDS is driven by immune activation and cytokine release with loss of integrity of the epithelial-endothelial interface resulting in alveolar and interstitial oedema, loss of pulmonary compliance, and impaired gas exchange [6]. The mainstay of therapy in ARDS is supportive treatment in the critical care environment [7]. Numerous clinical trials have studied pharmacological interventions in ARDS, but to date these have failed to demonstrate therapeutic benefit [8]. More recently, immunomodulatory therapies including dexamethasone and IL-6 antagonism have proven to be of benefit in mechanically ventilated patients with COVID-19, supporting the potential benefit of immunomodulation in critically ill patients with respiratory failure [9], [10], [11], [12].

Mesenchymal stromal cells (MSCs) have been proposed as a possible therapy for ARDS with pleiotropic immunomodulatory, reparative, and antimicrobial effects [13], [14], [15], [16], [17]. The mechanisms of action of MSCs include (a) paracrine secretion of growth factors, cytokines, and antimicrobial peptides [17], [18], [19], [20], [21], [22], [23], (b) direct cell contact transferring functional mitochondria to damaged cells and immune cells [24, 25], and (c) release of extracellular vesicles which can transfer mitochondria, mRNA and microRNA [26], [27], [28], [29], [30]. In pre-clinical models of ARDS, MSCs improve physiological outcomes, including oxygenation and lung compliance, and survival [[14], [15], [16], 31, 32]. In human ex vivo lung perfusion (EVLP) models, MSC administration effectively restored alveolar fluid clearance, a measure of the integrity of the alveolar epithelial-endothelial barrier [33], [34], [35]. Phase 1 and phase 2 clinical trials of bone marrow, adipose, and umbilical cord derived MSCs and MSC-like cells suggest these are safe in patients with ARDS but these trials were not powered to test clinical efficacy [36], [37], [38], [39], [40], [41], [42]. The optimal cell type, source, manufacturing method, and dose to use in patients with ARDS have also not been determined.

The REALIST trial investigates ORBCEL-C, a defined cellular product, consisting of CD362 enriched umbilical cord (UC)-derived MSCs. UC-derived MSCs have the advantage over bone marrow derived cells of both abundant source tissue and being readily obtained without risk to donor, making them both less expensive and safe (for donors). UC-derived MSCs have demonstrated comparable efficacy to bone marrow (BM)-derived MSCs in a clinically relevant model of ARDS induced by intratracheal *Escherichia Coli* administration [15]. CD362 is a heparan sulphate proteoglycan identified as a marker for MSC isolation and therapeutic development [43]. A defined subpopulation of MSCs offers an advantage in terms of purity of the cellular product, which may be more likely to fulfil emerging standards regarding cell isolation and characterisation. In preclinical models, CD362+ enriched MSCs isolated from UC-tissue were equally efficacious as traditionally manufactured plastic adherent MSCs in attenuating bacterial and ventilator induced lung injury [14, 16].

In this phase 1 study we tested the safety of a single intravenous infusion of ORBCEL-C in mechanically ventilated patients with moderate to severe ARDS, defined by the Berlin criteria. The maximum tolerated dose, over the range of 100 to 400 × 10^6^ cells, was determined.

## 2 Methods

### 2.1 Study design and participants

The REALIST phase 1 study was a UK multicentre (5 sites), open label, dose escalation trial in which cohorts of patients with moderate to severe ARDS, received increasing doses of a single intravenous infusion of ORBCEL-C in a 3 + 3 design [44]. Eligible patients were mechanically ventilated, within 48 h of the onset of moderate to severe ARDS, defined by Berlin criteria [1]. Inclusion and exclusion criteria are detailed in Table 1.

**Table 1:** Inclusion and exclusion criteria .

Inclusion criteria Moderate to severe ARDS as defined by the Berlin definition Onset within 1 week of identified insult Within the same 24 h time period Hypoxic respiratory failure (PaO_2_/FiO_2_ ratio ≤ 27 kPa on PEEP ≥ 5 cm H_2_0) Bilateral infiltrates on chest X-ray consistent with pulmonary oedema not explained by another pulmonary pathology Respiratory failure not fully explained by cardiac failure or fluid overload The patient is receiving invasive mechanical ventilation
Exclusion criteria More than 48 h from the onset of ARDS Age < 16 years Patient is known to be pregnant (pregnancy test to be performed in female patients of childbearing age) Participation in clinical trial of an investigational medicinal product within 30 days Major trauma in prior 5 days Presence of any active malignancy (other than non-melanoma skin cancer) that required treatment within the last year. WHO Class III or IV pulmonary hypertension Venous thromboembolism currently receiving anti-coagulation or within the past 3 months Currently receiving extracorporeal life support (ECLS) Severe chronic liver disease with Child-Pugh score > 12 DNAR (Do Not Attempt Resuscitation) order in place Treatment withdrawal imminent within 24 h Consent declined Prisoners Non-English speaking patients or those who do not adequately understand verbal or written information unless an interpreter is available Previously enrolled in the REALIST trial

The trial was approved by a UK research ethics committee (18/NE/0006) and the Medicines and Health products Regulatory Agency (MHRA, CTA 32485/0034/001–0001 and Eudract Number 2017–000584–33). The study was registered on clinicaltrials.gov (NCT03042143). The study protocol is available as a supplementary file (Supplemental file 1: **Protocol v 3.0 26.06.2019**). Patients or their relatives provided written informed consent.

Patients were treated with ascending doses of ORBCEL-C in a 3 + 3 design. Dose limiting toxicity (DLT, defined by the presence of a serious adverse reaction) was assessed at day 7. The DMEC convened after each group of 3 patients had been recruited to a given dose and had completed 7 days follow-up, to approve progression to the next dose. The study planned to recruit 3 cohorts of 3 patients/cohort, but up to 18 patients could be recruited according to the dose escalation procedure if DLT toxicity occurred. The planned dose escalation procedure was as follows. If no patient within a dose cohort experienced DLT, then the trial proceeded to the next dose. If one patient in the cohort demonstrated DLT, a further three subjects would be treated at the same dose level. This dose escalation procedure planned to continue until at least two patients among a cohort of three to six patients had DLT or until the maximum planned cell dose had been tested. As a safety precaution only one patient across all sites received the cell infusion at one time and no further patients received treatment in the 24 h following the completion of their infusion.

### 2.2 Procedures

The investigational medicinal product (IMP) in REALIST phase 1 was ORBCEL-C. CD362-enriched cells were harvested from umbilical cord and expanded under Good Medical Practice (GMP) conditions at the National Health Service Blood and Transplant (NHSBT) Birmingham. Cells were cryopreserved and shipped frozen to cell therapy facilities (CTFs) located in proximity to the clinical sites.

After informed consent, patients were allocated to receive either 100, 200 or 400 × 10^6^ cells of ORBCEL-C according to the dose escalation protocol. IMP was thawed and diluted in Plasma-Lyte 148 to a total volume of 200mls, according to the REALIST study specific standard operating procedure, at the site's CTF. Laboratory studies demonstrated > 70% viability of cells thawed and diluted in accordance with the study SOP at 6 h. Patients were administered an intravenous bolus of chlorphenamine 10 mg before administration of the IMP, to reduce any histamine-mediated effects of the DMSO in the cell cryopreservant [45]. IMP was administered over 30–90 min, and the infusion was completed within 6 h of the onset of the thaw process. All other aspects of care were according to standard critical care guidelines and at the discretion of the treating physician. Lung protective ventilation was the standard of care with a tidal volume of 6 ml/kg predicted body weight (PBW) [7].

Baseline data (day 0) were collected in the 24 h before IMP administration. Physiological and ventilatory parameters, along with temperature and vasopressor doses, were recorded immediately before IMP administration, every 15 min during infusion of the IMP, and every hour for the 5 h following IMP administration. Daily data were collected until day 14 (or death or ICU discharge if sooner), including Sequential Organ Failure Assessment (SOFA) score, temperature, ventilatory, and arterial blood gas parameters, use of adjunctive therapies, and clinical laboratory assessments. Vital status and adverse events were followed up to day 90. Patients were followed up for significant medical events, including death, at 1 year.

A focused set of biological markers was measured by ELISA (Duoset kits, R&D systems) in plasma collected at day 0, 4, 7, and 14. This included markers of systemic inflammatory response that are associated with outcome in ARDS (IL-6, IL-8 and IL-18), and markers of epithelial injury (Surfactant protein-D [SP-D]) and endothelial activation/injury (ICAM-1 and Angiopoietin-2 [Ang2]). Anti-HLA antibodies were measured in serum samples collected at day 0, and day 28, using Luminex antibody detection and single antigen bead methods (One Lambda LABScreen). HLA typing was performed on retained ORBCEL-C samples administered to patients who developed anti-HLA antibodies at day 28, to determine if donor specific antibody reactivity had occurred.

### 2.3 Adverse event reporting

REALIST phase 1 recruited patients were already critically ill. Events expected in the critically ill (examples include transient hypoxaemia, agitation, delirium, organ failure, nosocomial infections, skin breakdown, and gastrointestinal bleeding) were not reported as adverse events unless considered to be related to the IMP or unexpectedly severe or frequent.

The following pre-specified adverse events occurring within 6 h of the start of infusion were collected

• An increase in vasopressor dose greater than or equal to the following:
• Noradrenaline: 0·1 mcg/kg/min
• Adrenaline: 0·1 mcg/kg/min
• Commencement of any vasopressor including noradrenaline, adrenaline, vasopressin, phenylephrine, and dopamine
• New ventricular tachycardia, ventricular fibrillation or asystole
• New cardiac arrhythmia requiring cardioversion
• hypoxaemia requiring an increase in FiO_2_ of 0·2 or more and an increase in PEEP of 5 or more to maintain Sp0_2_ in the target range
• Clinical scenario consistent with transfusion incompatibility or transfusion related infection (e.g. urticaria, new bronchospasm).
• The following pre-specified adverse events occurring within 24 h of the start of infusion were collected:
• Any death
• Any cardiac arrest
• Temperatures recorded as > 38·5 °C or temperatures that are recorded as > 38·5 °C prior to study drug administration and have increased by ≥ 1 °C

As ORBCEL-C had not been administered to patients with ARDS previously, all adverse events considered by the site investigator to be related to IMP (thereby an adverse reaction, AR) were considered unexpected. All serious adverse events (SAEs) related to the IMP (thereby a serious adverse reaction, SAR) were considered to be a suspected unexpected serious adverse reaction (SUSAR).

### 2.4 Outcomes

The primary objective of this study was to determine the safety of a single intravenous infusion of ORBCEL-C and to define a safe dose for a subsequent phase 2 trial. The primary safety outcome was the incidence of serious adverse events. Adverse events, including pre-specified infusion related adverse events, are reported to day 90. Although this phase 1 trial was not designed to evaluate efficacy, the primary efficacy outcome reported was oxygenation index (OI) at day 7. OI, calculated as (mean airway pressure [cmH_2_O] x FiO_2_ x 100)/PaO_2_[*k*P*a*]) independently predicts outcome in ARDS [46]. Secondary outcomes reported included: OI at day 4 and 14; physiological indices of pulmonary function (respiratory compliance, driving pressure, and PaO_2_/FiO_2_ (PF) ratio) and organ failure measured by SOFA score on days 4, 7, and 14. Clinical outcome measures including extubation, reintubation, ventilator-free-days (VFDs) at day 28, duration of ventilation, length of ICU and hospital stay, as well as 28- and 90-day mortality are reported. Definitions of clinical outcomes are provided in the phase 1 statistical analysis plan, available as a supplemental file (Supplemental file 2: Phase 1 Statistical Analysis Plan). Exploratory outcomes including biological markers of systemic inflammatory response, epithelial and endothelial injury, indices of coagulation, and anti-HLA antibodies are reported. Additional exploratory outcomes detailed in our protocol (which covered the phase 1 and the subsequent phase 2 studies) that were not measured in this phase 1 trial included pulmonary markers of inflammation and cell injury (as bronchoalveolar lavage was not carried out during the phase 1 study) and cardiac function (as echocardiography was not routinely conducted during this phase 1 trial). These exploratory outcomes will be assessed in the subsequent phase 2 clinical trial. Survival status and significant medical events at 1 year are reported.

### 2.5 Statistical analysis

The primary analysis was descriptive and focused on serious adverse events. Pulmonary and non-pulmonary organ function, clinical outcomes, and exploratory outcomes are reported as descriptive analyses with mean (standard deviation, SD) or median [Interquartile range, IQR] (see Supplemental file 2: Phase 1 Statistical Analysis Plan). For pulmonary and non-pulmonary organ function, as data was not available at all specified timepoints, imputed data from the last observed value is also provided.

### 2.6 Role of funding source

The trial was funded by the 10.13039/100010269Wellcome Trust Health Innovation Challenge Fund [reference 106939/Z/15/Z] and sponsored by the Belfast Health and Social Care Trust. The funder had no role in the study design, conduct or analysis.

Orbsen Therapeutics Ltd. has granted a non-exclusive, trial-specific licence to the Cellular and Molecular Therapies Division of the National Health Service Blood and Transplant Service to manufacture ORBCEL-C to GMP standards for the REALIST trial. Orbsen Therapeutics Ltd. has had no role in the study design, data acquisition, data analysis or manuscript preparation.

## 3 Results

### 3.1 Participants

**Fig. 1:**
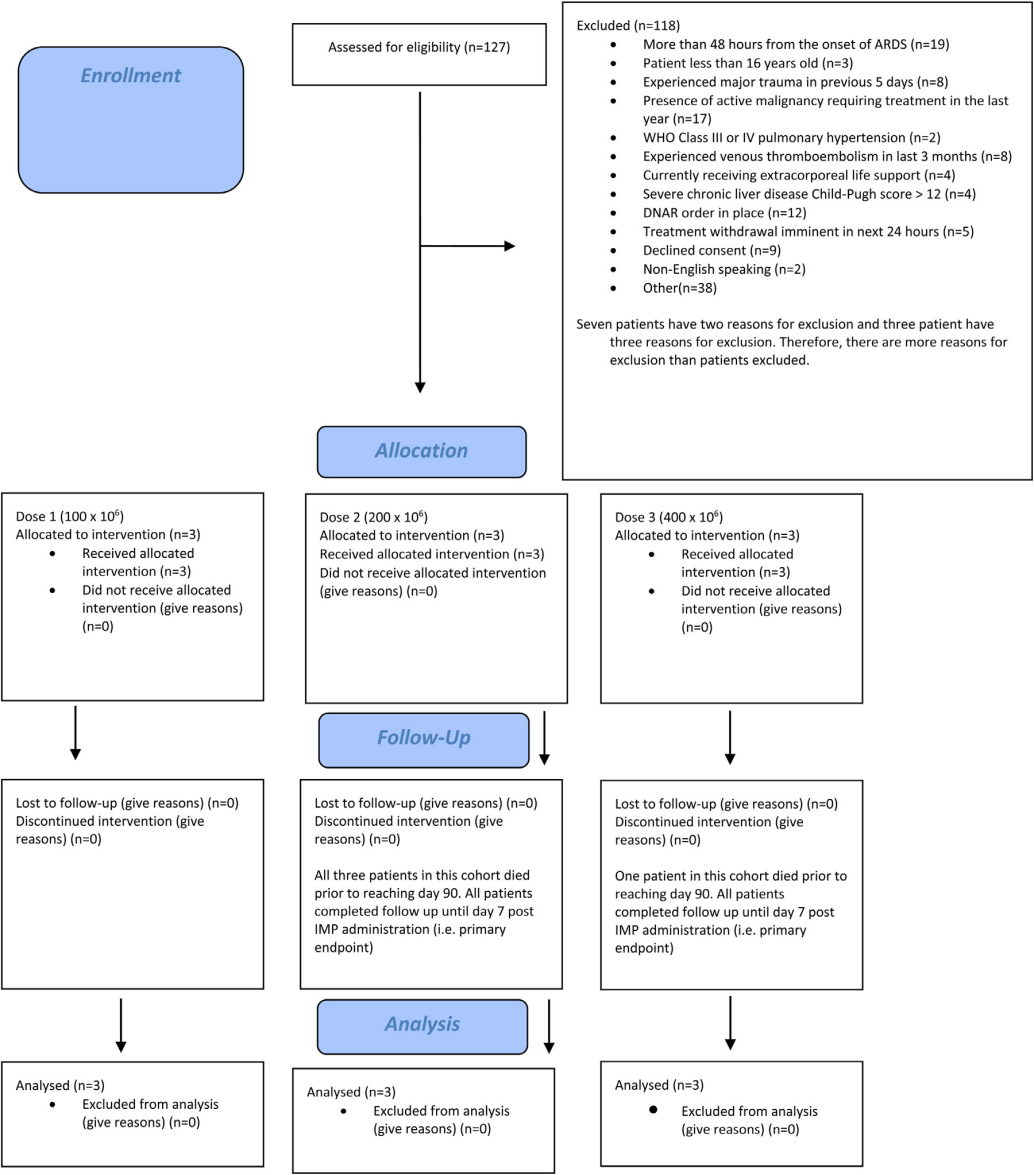
Consort diagram.

Nine patients were recruited between the 7th January 2019 and 14th January 2020, all from one of the participating sites: three patients per dose cohort (100 × 10^6^, 200 × 10^6^, and 400 × 10^6^ cells). 127 patients were assessed for eligibility across the five sites, of whom 118 were excluded with reasons for exclusion provided in Fig. 1 (Fig. 1: CONSORT diagram). All patients recruited completed infusion of the IMP and no patients were lost to follow up at day 90. Summary baseline characteristics for the included patients are described in Table 2. Individual baseline characteristics are described in Table 3.

**Table 2:** Summary baseline characteristics for each cohort.

	100 x 10^6^ cells	200 x 10^6^ cells	400 x 10^6^ cells	Total	
	*n* = 3	*n* = 3	*n* = 3	*n* = 9	
Gender Male Female	2(66·7%) 1(33·3%)	2(66·7%) 1(333%)	2(66·.7%) 1(33·3%)	6(66·7%) 3(33·3%)	
Age in years	58(14·2)	56(6·7)	47(20·1)	54(13·8)	
Temperature ( °C)	37·2(1·0)	37·6(2·1)	37·8(1·4)	37·5(1·4)	
Aetiology of ARDS*					
Gastric content aspiration	0(0·0%)	2(66·7%)	1(33·3%)	3(33·3%)	
Thoracic trauma	1(33·3%)	0(0·0%)	0(0·0%)	1(11·1%)	
Pneumonia	2(66·7%)	2(66·7%)	3(100·0%)	7(77·8%)	
Sepsis	2(66·7%)	2(66·7%)	2(66·7%)	6(66·7%)	
APACHE II Score	16·3(5·5)	25·0(2·6)	17·7(6·7)	19·7(6·1)	
Murray Lung Injury Score (LIS)	2·0(0·5)	3·1(0·6)	2·5(0·4)	2·5(0·6)	
First Qualifying PaO_2_/FiO_2_ Ratio [Min, Max]	16·6(3·6) [12·5, 18·9]	18·4(8·9) [8·1, 24]	23·6(1·4) [22·0, 24·8]	19·5(5·8) [8·1, 24·8]	
Worst PaO_2_/FiO_2_ ratio** [Min, Max]	21·7(8·2) [12·5,28·3]	12·7(4·9) [8·0,17·7]	21·2(3·6) [17·2,24·0]	18·5(6·7) [8·0,28·3]	
Total SOFA Score	12·0(4·2) *n* = 2	16·3(4·6)	10·3(3·2)	13·0(4·4) *n* = 8	
Oxygenation Index (cmH2O/kPa)	44·4(16·1) *n* = 2	133·7(141·0)	62·2(21·1)	84·6(86·9) *n* = 8	
Lowest Mean Arterial Pressure (mmHg)	62·0(3·6)	54·3(4·2)	61·3(6·1)	59·2(5·5)	
PEEP (cmH_2_O)	8·3(3·5)	12·3(2·5)	9·0(3·6)	9·9(3·4)	
Plateau Pressure (cmH_2_O)	17·5(6·4) *n* = 2	30·3(4·2)	25·7(1·5)	25·4(6·3) *n* = 8	
Driving Pressure (cmH_2_O)	9·0 (1·4) *n* = 2	18·0(2·6)	16·7(4·5)	15·3 (4·8) *n* = 8	
Mode of Ventilation					
SIMV	2(66·7%)	3(100·0%)	3(100·0%)	8(88·9%)	
PS	1(33·3%)	0(0·0%)	0(0·0%)	1(11·1%)	
Other	0(0·0%)	0(0·0%)	0(0·0%)	0(0·0%)	
None	0(0·0%)	0(0·0%)	0(0·0%)	0(0·0%)	
Tidal Volume (ml/kg PBW) [Min, Max]	6·2(0·6) [5·5, 6·7]	7·2(1·3) [5·8, 8·5]	6·7(1·0) [5·6, 7·4]	6·7(1·0) [5·5, 8·5]	
Adjuvant therapy at baseline (n,%)					
Neuromuscular blocking drugs	1(33·3%)	2(66·6%)	2(66·6%)	5(55·5%)	
APRV	0(0·0%)	0(0·0%)	0(0·0%)	0(0·0%)	
Nitric Oxide	0(0·0%)	2(66·6%)	1(33·3%)	3(33·3%)	
Prone positioning	0(0·0%)	0(0·0%)	0(0·0%)	0(0·0%)	
RRT	0(0·0%)	0(0·0%)	0(0·0%)	0(0·0%)	

Mean (SD) or Median [IQR] presented for continuous variables and no. (%) for all categorical variables.

*Patients may have > 1 aetiology of ARDS.

**Worst PF Ratio recorded on Day 0/24 h prior to randomisation.

ARDS = Acute Respiratory Distress Syndrome; APACHE = Acute Physiology and Chronic Health Evaluation; APRV = Airway Pressure Release Ventilation; SOFA = Sequential Organ Failure Assessment; PEEP = Positive End Expiratory Pressure; RRT = Renal Replacement Therapy.

**Table 3:** Baseline patient characteristics.

Patient	1	2	3	4	5	6	7	8	9

Treatment Dose	100 x 10^6^	100 x 10^6^	100 x 10^6^	200 x 10^6^	200 x 10^6^	200 x 10^6^	400 x 10^6^	400 x 10^6^	400 x 10^6^
Gender	Female	Male	Male	Female	Male	Male	Male	Female	Male
Age (years)	42	63	69	58	49	62	64	51	25
Temperature (°C)	38·1	37·5	36·1	36·2	36·7	40	36·4	39·2	37·7
ARDS aetiology	Sepsis	Thoracic trauma Pneumonia	Pneumonia Sepsis	Aspiration Pneumonia Sepsis	Aspiration	Pneumonia Sepsis	Pneumonia Sepsis	Pneumonia Sepsis	Aspiration Pneumonia
APACHE II Score	20	10	19	23	24	28	25	16	12
Murray Lung Injury Score (LIS)	2.5	2.0	1.5	2.8	3.8	2.8	2.3	3.0	2.3
First Qualifying PaO_2_/FiO_2_ Ratio	12·5	18·4	18·9	23·0	8·1	24·0	24·0	22·0	24·8
Worst PaO_2_/FiO_2_ ratio **	12·5	28·3	24·3	12·5	8·0	17·7	24·0	17·2	22·4
Total SOFA Score	15·0	9·0	–	19·0	19·0	11·0	14·0	9·0	8·0
Oxygenation Index (cmH_2_O/kPa)	55·8	–	33·0	59·1	296·3	45·6	47·6	86·4	52·7
Lowest Mean Arterial Pressure (mmHg)	59·0	66·0	61·0	59·0	51·0	53·0	56·0	68·0	60·0
PEEP (cmH_2_O)	12·0	8·0	5·0	10·0	15·0	12·0	5·0	10·0	12·0
Plateau Pressure (cmH_2_O)	22·0	–	13·0	29·0	35·0	27·0	26·0	27·0	24·0
Driving Pressure (cmH_2_O)	10·0	–	8·0	19·0	20·0	15·0	21·0	17·0	12·0
Mode of Ventilation	SIMV	PS	SIMV	SIMV	SIMV	SIMV	SIMV	SIMV	SIMV
Tidal Volume (ml/kg PBW)	6·3	6·7	5·5	7·2	8·5	5·8	7·0	7·4	5·6
Vital Status, Day of ICU Discharge or death	Alive, Day 24	Alive, Day 9	Alive, Day 9	Dead, Day 7	Dead, Day 14	Dead, Day 9	Dead, Day 8	Alive, Day 25	Alive, Day 11

*Patients may have > 1 aetiology of ARDS.

**Worst PF Ratio recorded on Day 0/24 h prior to randomisation.

ARDS = Acute Respiratory Distress Syndrome; APACHE = Acute Physiology and Chronic Health Evaluation; SOFA = Sequential Organ Failure Assessment; PEEP = Positive End Expiratory Pressure.

### 3.2 Safety outcomes and adverse events

Study drug infusion was generally well-tolerated with no adverse haemodynamic or respiratory physiological changes during infusion and for the 5 h following IMP administration (data not shown). Adverse events, including pre-specified infusion related events, are summarised in Table 4. In summary, eight adverse events were reported in four patients. Four adverse events, reported in three patients, were considered to be serious adverse events (SAEs). These SAEs were considered to be severe but were deemed unlikely to be, or not, related to study drug administration. Four adverse events were considered to be mild and possibly related to study drug administration therefore are categorised as adverse reactions (ARs). Two of these events, specifically pyrexia within 24 h of study drug administration, were reported as pre-specified infusion related adverse events. There was no dose limiting toxicity in any cohort and 400 × 10^6^ cells was determined to be the maximum tolerated dose.

**Table 4:** Adverse events and serious adverse events .

Patient	Dose Cohort	Description	Timing	Severity	Causality	Expectedness	Pre-specified adverse event	Classification
Non-serious adverse events								
1	100 x 10^6^	Pyrexia	Day 1 (< 24 h)	Mild (Grade 1)	Possibly	Unexpected	Yes	AR
1	100 x 10^6^	Non-sustained ventricular tachycardia	Day 1 (> 6 h)	Mild (Grade 1)	Possibly	Unexpected	No	AR
6	200 x 10^6^	Pyrexia	Day 1 (< 24 h)	Mild (Grade 1)	Possibly	Unexpected	Yes	AR
7	400 x 10^6^	Deranged LFTs	Day 1 (< 6 h)	Mild (Grade 1)	Possibly	Unexpected	No	AR
Serious adverse events								
1	100 x 10^6^	Perforated Duodenal Ulcer	Day 24	Severe (Grade 3)	Not related	N/A	No	SAE
1	100 x 10^6^	Myocarditis	Six weeks*	Severe (Grade 3)	Unlikely	N/A	No	SAE
6	200 x10^6^	Colonic Perforation	Day 9	Death (Grade 5)	Unlikely	N/A	No	SAE
8	400 x 10^6^	Bradycardia	Day 15	Severe (Grade 3)	Not related	N/A	No	SAE

*diagnosis on MRI at six weeks, for investigation of left ventricular systolic dysfunction during ICU admission.

In the lowest dose cohort, one patient experienced four adverse events (patient 1, Table 4). This patient was admitted with ARDS and sepsis due to *Streptococcus pneumoniae* and *influenzae A*. The patient developed pyrexia within 24 h of study drug administration which was reported as pre-specified infusion related event. Transient non-sustained ventricular tachycardia (NSVT) was reported on day 1, but not within the six hour window for reporting of pre-specified events. The NSVT resolved without haemodynamic compromise although the patient was commenced on an anti-arrhythmic. Both events were considered possibly related to the study drug. On day 24, this patient developed a perforated duodenal ulcer, requiring laparotomy and readmission to ICU. This was considered unrelated to study drug administration. Six weeks following study drug administration the patient underwent a cardiac MRI to investigate severe left ventricular systolic dysfunction noted during ICU admission. MRI findings were consistent with recent myocarditis, which was felt to have a viral aetiology and unlikely to be related to the IMP administration. The patient recovered and subsequent cardiology follow up at nine months after study drug administration found cardiac function had improved.

In the intermediate dose cohort, one patient experienced two adverse events (patient 6, Table 4). Pyrexia occurred within 24 h of study drug administration (reported as pre-specified infusion related event). On day 9 colonic perforation was demonstrated on computerised tomography (CT). The patient deteriorated clinically, with increased organ support requirements, was deemed too unstable for surgical intervention and subsequently died. Underlying decompensated alcoholic liver disease was felt to have contributed to the patients’ death. Of note, Child Pugh score at recruitment, was within eligible range. The colonic perforation was considered unlikely to be related to the study drug.

In the highest dose cohort, two patients experienced a single adverse event. One patient (patient 8, Table 4) had a bradycardic episode with brief loss of cardiac output requiring a short period (30 s) of cardiopulmonary resuscitation prior to return of spontaneous circulation and resolution of the event. This event occurred on day 15 and was considered to be unrelated. Another patient developed acute derangement of liver function tests within six hours of study drug administration (patient 7, Table 4). This was considered possibly related, however improved spontaneously over the following six days. This patient died on day 8 due to sequelae of their underlying disease (multiorgan failure due to intra-abdominal sepsis and strangulated hernia). The death was unrelated to study drug administration and the deranged liver function was not felt to have contributed.

### 3.3 Physiological and clinical outcomes

**Fig. 2:**
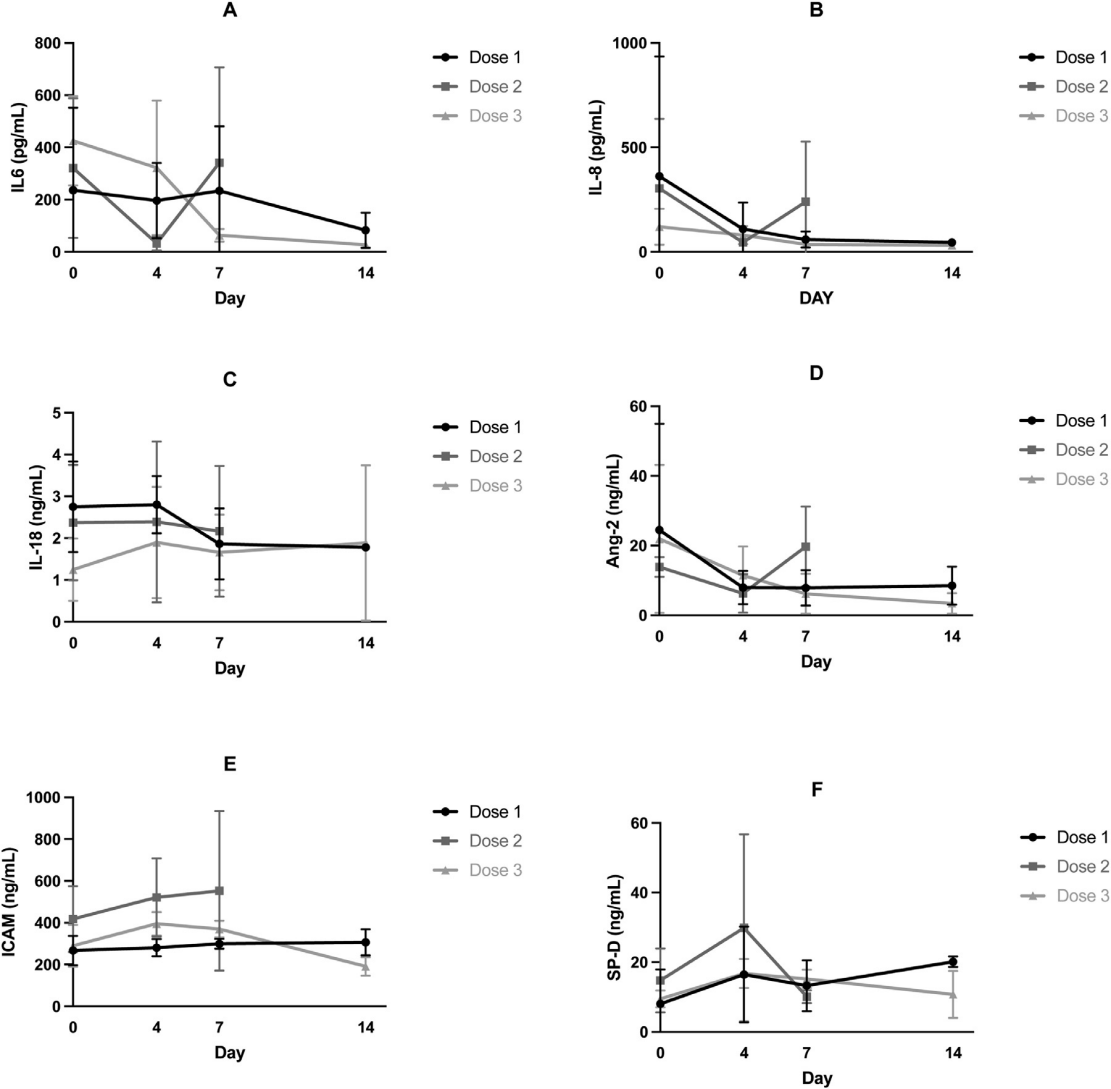
Pulmonary (Oxygenation Index, P/F ratio, Respiratory Compliance, Driving pressure) and non-pulmonary organ function (SOFA score) outcomes Mean (SD) are provided for each dose cohort (dose 1: 100 × 10 6; dose 2: 200 × 10 6; dose 3: 400 × 10 6) at day 0 (baseline), day 4, day 7 and day 14. (A) Oxygenation index (B) P/F Ratio (C) Respiratory Compliance (D) Driving Pressure (E) SOF A Score.

Measures of pulmonary and systemic organ function, including OI, PF ratio, respiratory compliance, driving pressure, and SOFA score until day 14 are presented in Fig. 2 and tabulated in Supplementary Table 1. Imputed data (using last observed value) for these outcomes are also provided in Supplementary Fig. 1 and Supplementary Table 1. There was no evidence of any dose dependent effects of MSCs on these physiological measures. Clinical outcomes and adjuvant therapies are reported in Table 5.

**Table 5:** Clinical outcomes.

	100 x 10^6^ cells	200 x 10^6^ cells	400 x 10^6^ cells	Total
	*n* = 3	*n* = 3	*n* = 3	*n* = 9
Time to 1st successful extubation (hours)	*n* = 3 151·5(166·3) 92·8[22·5339·1]	*n* = 0	*n* = 2 292·4(185·6) 292·4[161·1423·6]	*n* = 5 207·8(168·5) 161·1[92·8339·1]
Total number of reintubation after a planned extubation	2	0	0	2
Average number of reintubations per patient	0·7(1·2) 0[0,2]	0(0·0) 0[0,0]	0(0·0) 0[0,0]	0·2(0·7) 0[0,0]
Ventilation Free Days at day 28	15·7(13·7) 22[0,25]	0(0,0) 0[0,0]	9.0(9.5) 8[0,19]	8.2(10.8) 0[0,19]
Duration of Ventilation (days) ‡	8·3(6·8) 6·0[3·0,16·0]	9·0(3·6) 8·0[6·0,13·0]	12·0(7·0) 9·0[7·0,20·0]	9·8(5·5) 8·0[6·0,13·0]
Length of ICU stay (days)	*n* = 3 13·0(8·7) 8·0[8·0, 23·0]	*n* = 3 9·0(3·6) 8·0[6·0, 13·0]	*n* = 3 13·7(9·1) 10·0[7·0, 24·0]	*n* = 9 11·9(6·9) 8·0[8·0, 13·0]
Length of hospital stay (days)	*n* = 3 44·3(10·5) 44·0[34·0, 55·0]	*n* = 3 9·0(3·6) 8·0[6·0, 13·0]	*n* = 3 20·3(15·3) 17·0[7·0, 37·0]	*n* = 9 24·6(18·3) 17·0[8·0, 37·0]
28 day mortality †	0(0·0)	3(100·0%)	1(33·3%)	4(44·4%)
90 day mortality †	0(0·0)	3(100·0%)	1(33·3%)	4(44·4%)
Adjuvant therapy required between day 1 and day 14 (n,%)				
Neuromuscular blocking drugs	1(33·3%)	2(66·7%)	3(100·0%)	6(66·7%)
Nitric Oxide	0(0·0%)	2(66·7%)	3(100·0%)	5(55·6%)
APRV	0(0·0%)	1(33·3%)	0(0·0%)	1(11·1%)
ECMO or ECCO_2_R	0(0·0%)	1(33·3%)	0(0·0%)	1(11·1%)
Prone position	0(0·0%)	0(0·0%)	1(33·3%)	1(11·1%)
RRT	0(0·0%)	1(33·3%)	0(0·0%)	1(11·1%)

Mean (SD), median[IQR] or n(%) presented.

*Row percentages displayed.

†Percentages calculated based on total number of patients recruited to each group.

‡ Duration of ventilation is counted from time of study drug administration to being successfully free from assisted breathing.

ECMO = Extracorporeal membrane oxygenation; ECCO2R = Extracorporeal carbon dioxide removal; APRV = Airway Pressure Release Ventilation; RRT = Renal Replacement Therapy.

### 3.4 Biological and clinical laboratory measures

**Fig. 3:**
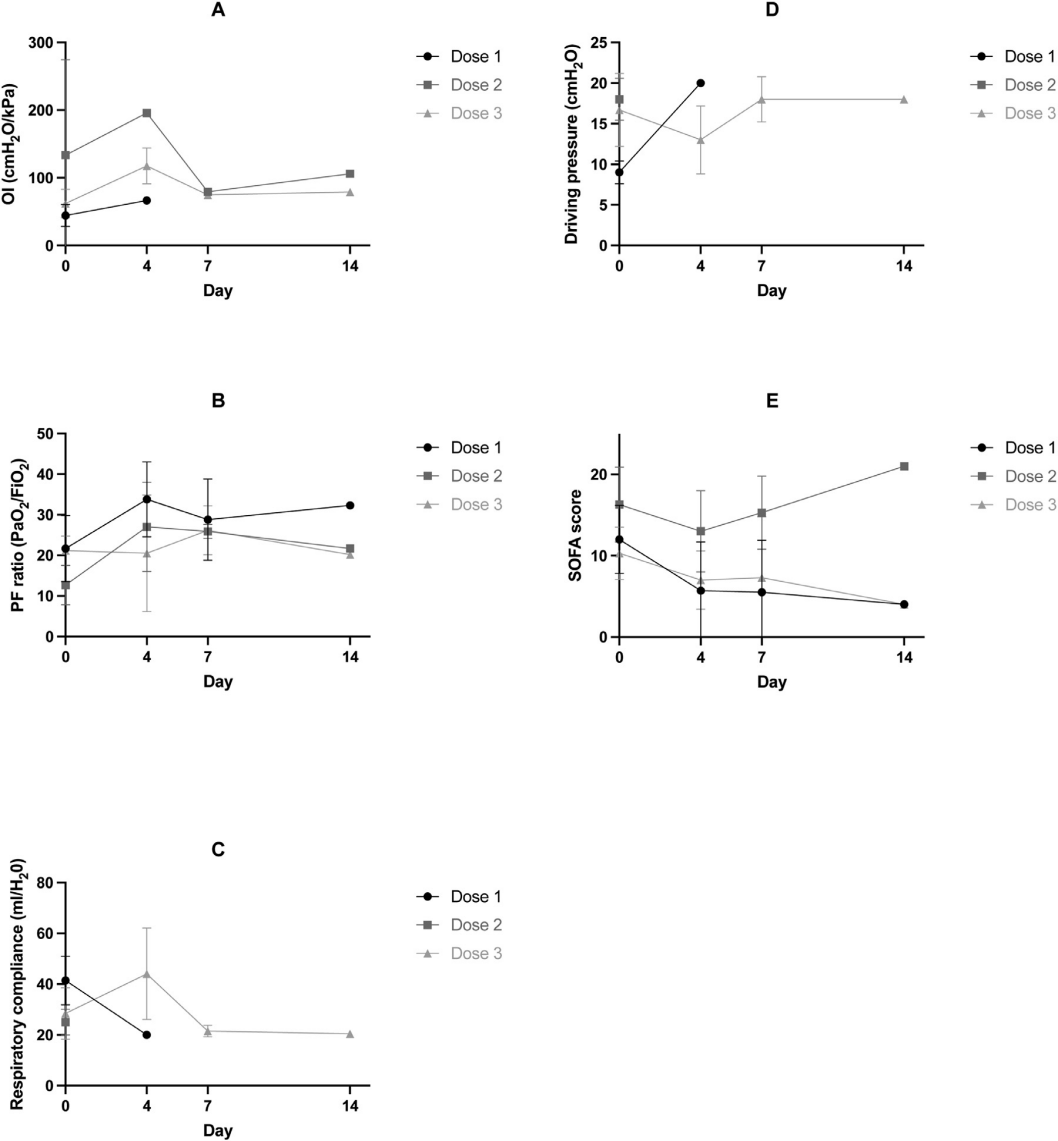
Plasma biological markers of systemic inflammatory response (IL-6, IL-8 and IL-18), markers of epithelial injury (Surfactant protein-D [SP-D]) and endothelial activation/injury (ICAM-1 and Angiopoietin 2 [Ang-2]). Mean (SD) are provided for each dose cohort (dose 1: 100 × 10 6; dose 2: 200 × 106; dose 3: 400 × 106) at day 0 (baseline), day 4, day 7 and day 14. A) IL-6 B) IL-8 C) IL-18 D) Ang-2 E) ICAM F) SP-D.

We measured plasma markers of systemic inflammation (IL-6, IL-8, and IL-18), epithelial cell injury (SP-D) and endothelial injury /activation (Ang-2/ICAM-1), which have all been shown to be elevated in patients with ARDS. There were no important trends over time (from baseline to day 4, 7 and 14) identified, and there were no apparent dose dependent effect of MSCs on any of these markers (Fig. 3). Individual patient biomarker measurements are included in the supplement (Supplementary Table 2). Clinical laboratory data (including CRP, renal indices, indices of coagulation, haemoglobin or leucocytes) following study drug administration did not show any trends or signals of harm in relation to the study drug administration (Supplemental Table 3).

### 3.5 Anti-HLA antibodies

Baseline (*n* = 9) and day 28 (*n* = 4) serum samples were analysed for anti-HLA antibodies (Supplemental Table 4). At day 28 samples were not available for four patients who had died, and one patient was unable to provide a sample. No patients had anti-HLA antibodies at baseline, and of the four patient samples analysed at day 28, two developed anti-HLA antibodies (patient 1 and patient 8). One of these patients (patient 1) had antibody reactivity towards one antigen found on HLA typing of the donor ORBCEL-C infusion. However, the HLA antigen (HLA-A*01) is common [47], and as the patient had been transfused blood products between day 0 to 28, they may have been exposed to this antigen during transfusion. This patient also had HLA-antibody reactivity towards HLA-B antigens which were not accounted for by the HLA type of the donor ORBCEL-C infusion. None of the HLA-antibodies developed by patient 8 matched the HLA type of the donor ORBCEL-C infusion.

### 3.6 Survival and long term follow up

Four patients died within 28 days of study drug administration (Table 5, day 28 mortality 44%). All patients within the intermediate dosing cohort had died before day 28. One patient in this intermediate dose cohort was transferred to a different unit for ECMO therapy on day 2 due to refractory hypoxaemia and subsequently died on day 14 due to multiorgan failure (patient 5). Another patient in the intermediate dose cohort died on day 7 due to multiorgan failure as sequelae of pulmonary aspiration (patient 4). The death of the third patient in the intermediate dose cohort (patient 6) and one patient in the highest dose cohort (patient 7) are described earlier. Each death was reviewed in detail, and none were felt to be related to study drug administration. No further patients had died at day 90 following study drug administration. All surviving patients have been followed up to one year and none had died by this time point. One patient (patient 2) has had two significant medical events necessitating hospital admissions (1) mechanical fall with a spinal fracture (2) acute stroke. The remaining surviving patients have had no significant medical events reported at follow up.

## 4 Discussion

In this phase 1 trial, CD362 enriched human umbilical cord derived MSCs (ORBCEL-C) were well tolerated in patients with moderate to severe ARDS. No severe adverse events related to study drug administration or dose limiting toxicity were reported in any dose cohort (100, 200 or 400 × 10^6^ cells). Adverse reactions considered possibly related to study drug administration included pyrexia, non-sustained ventricular tachycardia, and deranged liver function. These events occurred early after study drug administration therefore a relationship could not be excluded, but this was a critically ill population in whom these events may have been related to their underlying condition. Follow up of patients to one year following MSC administration has not identified safety concerns. These findings support the safety of intravenous administration of ORBCEL-C, up to a dose of 400 × 10^6^ cells, in patients with moderate to severe ARDS.

The emerging safety profile of MSC therapy in critically ill patients with ARDS, is supported by the findings of other MSC clinical trials. A recent systematic review of intravascular MSC therapy for a range of clinical conditions demonstrated MSCs compared to control therapy was associated with an increased risk of fever, but there was no association with non-fever infusional toxicity, infection, thrombotic embolic events, death or malignancy [48]. In other trials of MSCs in ARDS, no infusion related toxicity or serious adverse events related to MSC administration has been reported [36], [37], [38], [39], [40], [41], [42]. There has been variation in dosing regimens and tissue sources of MSCs investigated in ARDS. Matthay and co-investigators, in the START phase 1 trial, administered a single intravenous infusion of either 1, 5, or 10 × 10^6^ BM-derived cells/kg PBW and in the absence of safety concerns proceeded to investigate a single intravenous infusion of 10 × 10^6^ cells/kg PBW in their phase 2 trial [37, 38]. Yip et al. investigated the same dose regimen in a phase 1 dose escalation study of UC-derived MSCs [40]. The MUSTARDS trial investigated administration of a single intravenous infusion of either 300 or 900 × 10^6^ cells/dose of Multistem (BM-derived multipotent adult progenitor cells, BM-MAPC) in their phase 1 trial, and proceeded with the higher dosing regimen in phase 2 [39]. Zheng et al., investigated a single intravenous infusion of 1 × 10^6^ adipose-derived MSCs in a small RCT. [36] Lv et al., investigated a single intravenous infusion of 1 × 10^6^ UC-derived MSCs in a single arm open label study [49].

These trials have not convincingly demonstrated dose dependent effects, despite evidence of dose-dependent effects in preclinical investigations in ARDS [16, 50]. In START phase 1 numerically greater improvements in lung injury score and SOFA score were reported in the highest dose cohorts, but the sample size in each dose cohort was small (*n* = 3) and differences were not statistically significant [37]. In a study of healthy male volunteers, MSC administration in a model of lipopolysaccharide (LPS) induced systemic inflammation demonstrated dose dependent adverse effects at 4 × 10^6^ cells/kg compared to lower doses of 1 × 10^6^ cells/kg, 0·25 × 10^6^ cells/kg or placebo [51]. These included an enhanced febrile response and a transient increase in markers of coagulation activation, however this did not translate into clinically relevant thromboembolic events [51]. In our trial standardised doses were used rather than doses per body weight to support the feasibility of manufacture and delivery of the MSC product. The doses chosen (100, 200, and 400 × 10^6^) are equivalent to approximately 1·5, 3, and 6 × 10^6^ cells/kg respectively, for an average adult with PBW of 70 kg. Lower maximum doses were chosen, compared to other studies of MSCs in ARDS, in light of the evidence of dose dependent coagulation activation in the healthy human LPS model [51]. We did not observe any dose dependent effects in either physiological markers of lung or systemic organ dysfunction nor in biological markers of inflammation or cell injury. However, in our phase 1 study, there was no placebo group to allow for suggestion of efficacy and with *n* = 3 per group there is no power to show significant differences between dose cohorts.

Efficacy of MSC therapy in patients with ARDS has yet to be determined and clinical trials to date have been underpowered to report clinical outcomes. In the MUSTARDS phase 2 randomised placebo controlled trial of MultiStem in 30 patients with ARDS, a possible improvement in clinical outcomes was reported (day 28 mortality 25% vs 40%; VFD 12·9 vs 9·2; ICU free days 10·3 vs 8·9) [39]. However the full peer reviewed report is awaited, and conclusions about efficacy must be guarded given the study size. Matthay et al., reported no significant difference in 28-day mortality (30% MSC group vs 15% placebo group, odds ratio 2·4, 95% CI 0·5 to 15·1) in their 60 patient START phase 2 randomised, placebo-controlled trial of bone marrow-derived MSC therapy using a dose of 10 × 10^6^ cells/kg [38]. In this study cell viability was measured post thaw and found to be less than expected in some cases (range 36 to 85%). Interestingly in a post-hoc analysis the authors found that patients treated with higher viability cells appeared to have improved OI, and lower mortality compared with those receiving cells with lower viability. However, this post-hoc analysis involved very small numbers and the findings need to be validated in a larger study. A procedure to wash the dimethyl sulfoxide (DMSO) containing cryoprotectant from the final cellular product before administration was implicated in loss of cell viability. In response to this, cells for the REALIST study underwent cryopreservation in a lower concentration of DMSO and in human albumin-containing cryopreservant. After thaw the cryopreserved cells were added directly to crystalloid solution, with no wash step, and patients were pre-treated with an antihistamine to counter any DMSO related effects [45]. Validation work during manufacture of the cellular product confirmed ORBCEL-C infusions had a post thaw cell viability > 70% for at least 6 h following the thaw procedure.

Biological markers of inflammation (IL-6, IL-8, and IL-18), and of epithelial (SP-D) and endothelial (Ang-2/ICAM-1) cell injury, were evaluated as part of the exploratory analysis in this REALIST phase 1 study. These biological markers are elevated in ARDS and predictive of poorer clinical outcomes [52], [53], [54], [55], [56]. In this REALIST phase 1 study we do not demonstrate any important trends in these biological markers over time following MSC administration. The small numbers, and absence of control group for comparison, limit any conclusions which can be drawn. Other investigators have reported positive findings in relation to biological markers following MSC administration. In the small RCT (*n* = 5 in each group) by Zheng et al., plasma SP-D concentration reduced significantly from baseline to day 5 following MSC administration, and plasma IL-6 and IL-8 numerically reduced from baseline to day 5, however there was no significant differences compared to the placebo group [36]. Similarly, in the START phase 1 study the median concentration of biomarkers (IL-6, IL-8, Ang-2 and RAGE (receptor for advanced glycation end-products)) reduced from baseline to day 3 following MSC administration, but there was no placebo group for comparison [37]. In the START phase 2 trial, a post-hoc analysis demonstrated patients treated with higher viability cells had a greater fall in Ang-2 concentrations [38]. Importantly, in a subsequent report of biomarkers from bronchoalveolar lavage (BAL) at 48 h in the START phase 2 trial (*n* = 17 MSC group, *n* = 10 placebo group), MSC administration significantly reduced BAL Ang-2, IL-6 and TNF receptor-1 concentrations compared to placebo [57]. These biological markers will be assessed in our phase 2 trial.

MSCs are considered to be relatively immune evasive, lacking MHC Class II antigen expression on their surface [58]. As part of the exploratory analysis in the REALIST phase 1 study, we report the development of anti-HLA antibodies in two patients after MSC administration, one of whom developed donor specific anti-HLA antibodies. Development of anti-HLA antibodies following MSC infusion has been evaluated in clinical trials in other conditions [59], [60], [61], [62], [63], though development of donor specific anti-HLA antibodies has been rarely reported [60, 61, 63]. The incidence of developing anti-HLA antibodies in critically ill patients is unknown, and these patients may have other sensitising events, such as blood transfusions, which can also lead to the development of anti-HLA antibodies. Immunological responses to MSC administration in ARDS will be evaluated further in Phase 2.

In this trial there was a 44% in a cohort of patients with moderate to severe ARDS. This is towards the upper range of mortality previously been reported in this population [4]. In a systematic review and meta-analysis of control arm mortality in randomised controlled trials in ARDS, when the inclusion criteria included a PF ratio consistent with moderate to severe ARDS, the control arm mean mortality rate was 35·1% [5]. The mortality rate in this phase 1 study is consistent with the baseline severity of illness. In the intermediate dose cohort for example, patients had a mean APACHE II score of 25 which is known to predict mortality rates greater than 50% [64]. No deaths in the study were deemed to be related to MSC infusion, and as a single arm study with a small sample size no conclusions can be made regarding the impact of MSC infusion on mortality. Limited reports of long-term follow up after MSC administration in ARDS have not raised safety concerns [38, 42, 65]. Our trial is the first to conduct long term follow up for significant medical events, as well as mortality, and supports the long-term safety profile of ORBCEL-C in this patient population.

In conclusion, the REALIST phase 1 trial study has shown that administration of a single intravenous infusion of ORBCEL-C, up to 400 × 10^6^ cells, is safe and feasible in critically ill patients with moderate to severe ARDS. Based on the absence of dose limiting toxicity or safety concerns in this phase 1 trial, a dose of 400 × 10^6^ cells has been approved as the intervention for the planned phase 2 randomised placebo-controlled REALIST trial (NCT03042143). This phase 2 trial will assess efficacy of MSC therapy in ARDS and, in light of the COVID-19 pandemic, will also evaluate MSC therapy in a separate cohort of patients with ARDS due to COVID-19 [66].

## Declaration of Competing Interest

EG receives funding by the Wellcome Trust Health Innovation Challenge Fund [reference 106939/Z/15/Z] for the described work. COK, DMcA, JL, JS & AK are investigators on the grant funding this work from Wellcome Trust Health Innovation Challenge Fund [reference 106939/Z/15/Z]. DMcA, COK & MC are investigators on a grant received from the Northern Ireland Health and Social Care Research and Development Division to fund an additional COVID-19 cohort during phase 2 of this study**.** JL reports consulting fees from Baxter Healthcare and GlaxoSmithKline, provision of medicolegal reports to the Irish Clinical Indemnity Scheme, participation in DSMB for other clinical trials and is named on a patent for InspireShield, a device to reduce COVID-19 spread. AK reports grants received from MRC UK (MR/R025096/1 and MR/S009426/1) and a HORIZON 2020 MSC individual fellowship. MSH is supported by a NIHR Clinician Scientist Fellowship (CS-2016–16–011). GDP is supported by the 10.13039/100005622Health Research (NIHR) Applied Research Collaboration (ARC) West Midlands and is a director of research for the Intensive Care Society. GC reports grants from Health Research Board (Ireland) Emerging Clinician Scientist Award and the United States Department of defense Discovery Award. DMcA reports grants from NIHR, Innovate UK, MRC and Novavax as an investigator in ARDS and COVID-19 studies. DMcA reports consultancy fees unrelated to this work from Bayer, GlaxoSmithKline, Boehringer Ingelheim**,** Novartis and Eli Lilly. DMcA reports payments from GlaxoSmithKline as an educational seminar speaker. DMcA is a member of the DSMB for Vir Biotechnology, Inc and Faron Pharmaceuticals. DMcA has a patent for a novel treatment for inflammatory disease. DMcA is a director of research for the Intensive Care Society and Director of the EME programme for MRC/NIHR. DMcA reports a spouse who has received consultancy fees from INSMED and from the California Institute for Regenerative unrelated to this clinical trial. COK has received consultancy fees from INSMED, unrelated to this work, and fees for participation in grant panels for the Californian Institute of Regenerative Medicine, unrelated to this clinical trial. COK reports a spouse who has received grants from NIHR, Innovate UK, MRC and Novavax. COK reports a spouse who has received consultancy fees unrelated to this work from Bayer, GlaxoSmithKline, Boehringer Ingelheim, Novartis and Eli Lilly. COK reports a spouse who has received payments from GlaxoSmithKline as an educational seminar speaker. COK reports a spouse who is a member of the DSMB for Vir Biotechnology, Inc and Faron Pharmaceuticals. COK reports a spouse who has a patent for a novel treatment for inflammatory disease. COK reports a spouse who is a director of research for the Intensive Care Society and Director of the EME programme for MRC/NIHR. All other authors report no declarations of interest. The views expressed in this publication are those of the authors and not necessarily those of the National Health Service, the National Institute for Health Research (NIHR), or the Department of Health and Social Care.

## Appendix Supplementary materials

Image, application 1

Image, application 2

Image, application 3

Image, application 4

Image, application 5

Image, application 6

Image, application 7

Image, application 8

## References

[1] Ranieri V.M., Rubenfeld G.D., Thompson B.T. (2012). Acute respiratory distress syndrome: the Berlin definition. JAMA.

[2] Herridge M.S., Tansey C.M., Matte A. (2011). Functional disability 5 years after acute respiratory distress syndrome. N Engl J Med.

[3] Bienvenu O.J., Friedman L.A., Colantuoni E. (2018). Psychiatric symptoms after acute respiratory distress syndrome: a 5-year longitudinal study. Intensive Care Med.

[4] Bellani G., Laffey J.G., Pham T. (2016). Epidemiology, patterns of care, and mortality for patients with acute respiratory distress syndrome in intensive care units in 50 countries. JAMA.

[5] Saha R., Assouline B., Mason G., Douiri A., Summers C., Shankar-Hari M. (2021). Impact of differences in acute respiratory distress syndrome randomised controlled trial inclusion and exclusion criteria: systematic review and meta-analysis. Br J Anaesth.

[6] MacSweeney R.M., McAuley D.F. (2016). Acute respiratory distress syndrome. Lancet.

[7] Griffiths M.J.D., McAuley D.F., Perkins G.D. (2019). Guidelines on the management of acute respiratory distress syndrome. BMJ Open Resp Res.

[8] Shaw T.D., McAuley D.F., O'Kane C.M (2019). Emerging drugs for treating the acute respiratory distress syndrome. Expert Opin Emerg Drugs.

[9] Horby P., Lim W.S., Emberson J.R. (2021). Dexamethasone in hospitalized patients with COVID-19. N Engl J Med.

[10] (2020). Effect of hydrocortisone on mortality and organ support in patients with severe COVID-19: the REMAP-CAP COVID-19 corticosteroid domain randomized clinical trial. JAMA.

[11] (2021). Interleukin-6 receptor antagonists in critically Ill Patients with COVID-19. N Engl J Med.

[12] (2021). Tocilizumab in patients admitted to hospital with COVID-19 (RECOVERY): a randomised, controlled, open-label, platform trial. Lancet.

[13] Devaney J., Horie S., Masterson C. (2015). Human mesenchymal stromal cells decrease the severity of acute lung injury induced by E. coli in the rat. Thorax.

[14] Masterson C., Devaney J., Horie S. (2018). Syndecan-2-positive, bone marrow-derived human mesenchymal stromal cells attenuate bacterial-induced acute lung injury and enhance resolution of ventilator-induced lung injury in rats. Anesthesiology.

[15] Curley G.F., Jerkic M., Dixon S. (2017). Cryopreserved, Xeno-free human umbilical cord mesenchymal stromal cells reduce lung injury severity and bacterial burden in rodent escherichia coli-induced acute respiratory distress syndrome. Crit Care Med.

[16] Horie S., Masterson C., Brady J. (2020). Umbilical cord-derived CD362(+) mesenchymal stromal cells for E. coli pneumonia: impact of dose regimen, passage, cryopreservation, and antibiotic therapy. Stem Cell Res Ther.

[17] Krasnodembskaya A., Song Y., Fang X. (2010). Antibacterial effect of human mesenchymal stem cells is mediated in part from secretion of the antimicrobial peptide LL-37. Stem Cells.

[18] Zhu H., Xiong Y., Xia Y. (2017). Therapeutic effects of human umbilical cord-derived mesenchymal stem cells in acute lung injury mice. Sci Rep.

[19] Sung D.K., Chang Y.S., Sung S.I., Yoo H.S., Ahn S.Y., Park W.S. (2016). Antibacterial effect of mesenchymal stem cells against Escherichia coli is mediated by secretion of beta- defensin- 2 via toll- like receptor 4 signalling. Cell Microbiol.

[20] Gupta N., Krasnodembskaya A., Kapetanaki M. (2012). Mesenchymal stem cells enhance survival and bacterial clearance in murine Escherichia coli pneumonia. Thorax.

[21] Lee J.W., Fang X., Gupta N., Serikov V., Matthay M.A. (2009). Allogeneic human mesenchymal stem cells for treatment of E. coli endotoxin-induced acute lung injury in the *ex vivo* perfused human lung. Proc Natl Acad Sci U S A.

[22] Fang X., Neyrinck A.P., Matthay M.A., Lee J.W. (2010). Allogeneic human mesenchymal stem cells restore epithelial protein permeability in cultured human alveolar type II cells by secretion of angiopoietin-1. J Biol Chem.

[23] Nemeth K., Leelahavanichkul A., Yuen P.S. (2009). Bone marrow stromal cells attenuate sepsis via prostaglandin E(2)-dependent reprogramming of host macrophages to increase their interleukin-10 production. Nat Med.

[24] Jackson M.V., Morrison T.J., Doherty D.F. (2016). Mitochondrial transfer via tunneling nanotubes is an important mechanism by which mesenchymal stem cells enhance macrophage phagocytosis in the *in vitro* and *in vivo* models of ARDS. Stem Cells.

[25] Islam M.N., Das S.R., Emin M.T. (2012). Mitochondrial transfer from bone-marrow-derived stromal cells to pulmonary alveoli protects against acute lung injury. Nat Med.

[26] Morrison T.J., Jackson M.V., Cunningham E.K. (2017). Mesenchymal stromal cells modulate macrophages in clinically relevant lung injury models by extracellular vesicle mitochondrial transfer. Am J Respir Crit Care Med.

[27] Zhu Y.G., Feng X.M., Abbott J. (2014). Human mesenchymal stem cell microvesicles for treatment of Escherichia coli endotoxin-induced acute lung injury in mice. Stem Cells.

[28] Song Y., Dou H., Li X. (2017). Exosomal miR-146a contributes to the enhanced therapeutic efficacy of interleukin-1β-primed mesenchymal stem cells against sepsis. Stem Cells.

[29] Silva J.D., Su Y., Calfee C.S. (2020). MSC extracellular vesicles rescue mitochondrial dysfunction and improve barrier integrity in clinically relevant models of ARDS. Eur Respir J.

[30] Fergie N., Todd N., McClements L., McAuley D., O'Kane C., Krasnodembskaya A. (2019). Hypercapnic acidosis induces mitochondrial dysfunction and impairs the ability of mesenchymal stem cells to promote distal lung epithelial repair. FASEB Journal.

[31] McIntyre L.A., Moher D., Fergusson D.A. (2016). Efficacy of mesenchymal stromal cell therapy for acute lung injury in preclinical animal models: a systematic review. PLoS ONE.

[32] Asmussen S., Ito H., Traber D.L. (2014). Human mesenchymal stem cells reduce the severity of acute lung injury in a sheep model of bacterial pneumonia. Thorax.

[33] Lee J.W., Fang X., Gupta N., Serikov V., Matthay M.A. (2009). Allogeneic human mesenchymal stem cells for treatment of E. coli endotoxin-induced acute lung injury in the *ex vivo* perfused human lung. Proc Natl Acad Sci U S A.

[34] Lee J.W., Krasnodembskaya A., McKenna D.H., Song Y., Abbott J., Matthay M.A. (2013). Therapeutic effects of human mesenchymal stem cells in *ex vivo* human lungs injured with live bacteria. Am J Respir Crit Care Med.

[35] McAuley D.F., Curley G.F., Hamid U.I. (2014). Clinical grade allogeneic human mesenchymal stem cells restore alveolar fluid clearance in human lungs rejected for transplantation. Am J Physiol Lung Cell Mol Physiol.

[36] Zheng G., Huang L., Tong H. (2014). Treatment of acute respiratory distress syndrome with allogeneic adipose-derived mesenchymal stem cells: a randomized, placebo-controlled pilot study. Respir Res.

[37] Wilson J.G., Liu K.D., Zhuo H. (2015). Mesenchymal stem (stromal) cells for treatment of ARDS: a phase 1 clinical trial. Lancet Resp Med.

[38] Matthay M.A., Calfee C.S., Zhuo H. (2019). Treatment with allogeneic mesenchymal stromal cells for moderate to severe acute respiratory distress syndrome (START study): a randomised phase 2a safety trial. Lancet Resp Med.

[39] Bellingan G, Jacono F, Bannard-Smith J (2019). Primary analysis of a Phase 1/2 study to assess MultiStem cell therapy, a regenerative advanced therapy medicinal product (ATMP), in acute respiratory distress syndrome (MUST-ARDS). B14 LATE BREAKING CLINICAL TRIALS: American Thoracic Society. Am J Respir Crit Care Med.

[40] Yip H.K., Fang W.F., Li Y.C. (2020). Human umbilical cord-derived mesenchymal stem cells for acute respiratory distress syndrome. Crit Care Med.

[41] Lv H., Chen W., Xiang A.P., Zhang Q., Yang Y., Yi H. (2020). Mesenchymal stromal cells as a salvage treatment for confirmed acute respiratory distress syndrome: preliminary data from a single-arm study. Intensive Care Med.

[42] Chen J., Hu C., Chen L. (2020). Clinical study of mesenchymal stem cell treatment for acute respiratory distress syndrome induced by epidemic influenza a (H7N9) infection: a hint for COVID-19 treatment. Engineering (Beijing, China).

[43] Devarapu S.K., Junhui X., Darisipudi M., Rocanin Arjo A., Anders H.J (2015). FP456CD362+ mesenchymal stem cell treatment of kidney disease in type 2 diabetic LEPR DB/DB mice. Nephrol Dial Transplant.

[44] Cook N., Hansen A.R., Siu L.L., Abdul Razak A.R (2015). Early phase clinical trials to identify optimal dosing and safety. Mol Oncol.

[45] Kollerup Madsen B., Hilscher M., Zetner D., Rosenberg J (2018). Adverse reactions of dimethyl sulfoxide in humans: a systematic review. F1000 Res.

[46] Seeley E., McAuley D.F., Eisner M., Miletin M., Matthay M.A., Kallet R.H. (2008). Predictors of mortality in acute lung injury during the era of lung protective ventilation. Thorax.

[47] Burt C., Cryer C., Fuggle S., Little A.M., Dyer P., HLA-A B. (2013). DR allele group frequencies in 7007 kidney transplant list patients in 27 UK centres. Int J Immunogenet.

[48] Thompson M., Mei S.H.J., Wolfe D. (2020). Cell therapy with intravascular administration of mesenchymal stromal cells continues to appear safe: an updated systematic review and meta-analysis. EClin Med.

[49] Lv H., Chen W., Xiang A.P., Zhang Q., Yang Y., Yi H. (2020). Mesenchymal stromal cells as a salvage treatment for confirmed acute respiratory distress syndrome: preliminary data from a single-arm study. Intensive Care Med.

[50] Jerkic M., Gagnon S., Rabani R. (2020). Human umbilical cord mesenchymal stromal cells attenuate systemic sepsis in part by enhancing peritoneal macrophage bacterial killing via heme oxygenase-1 induction in rats. Anesthesiology.

[51] Perlee D., van Vught L.A., Scicluna B.P. (2018). Intravenous infusion of human adipose mesenchymal stem cells modifies the host response to lipopolysaccharide in humans: a randomized, single-blind, parallel group, placebo controlled trial. Stem Cells.

[52] Ware L.B., Koyama T., Billheimer D.D. (2010). Prognostic and pathogenetic value of combining clinical and biochemical indices in patients with acute lung injury. Chest.

[53] Dolinay T., Kim Y.S., Howrylak J. (2012). Inflammasome-regulated cytokines are critical mediators of acute lung injury. Am J Respir Crit Care Med.

[54] Eisner M.D., Parsons P., Matthay M.A., Ware L., Greene K. (2003). Plasma surfactant protein levels and clinical outcomes in patients with acute lung injury. Thorax.

[55] Wada T., Jesmin S., Gando S. (2013). The role of angiogenic factors and their soluble receptors in acute lung injury (ALI)/acute respiratory distress syndrome (ARDS) associated with critical illness. J. Inflamm..

[56] Calfee C.S., Eisner M.D., Parsons P.E. (2009). Soluble intercellular adhesion molecule-1 and clinical outcomes in patients with acute lung injury. Intensiv Care Med.

[57] Wick K.D., Leligdowicz A., Zhuo H., Ware L.B., Matthay M.A. (2021). Mesenchymal stromal cells reduce evidence of lung injury in patients with ARDS. JCI Insight.

[58] Le Blanc K., Tammik C., Rosendahl K., Zetterberg E., Ringden O (2003). HLA expression and immunologic properties of differentiated and undifferentiated mesenchymal stem cells. Exp Hematol.

[59] Skyler J.S., Fonseca V.A., Segal K.R., Rosenstock J. (2015). Allogeneic mesenchymal precursor cells in type 2 diabetes: a randomized, placebo-controlled, dose-escalation safety and tolerability pilot study. Diabetes Care.

[60] Hare J.M., Fishman J.E., Gerstenblith G. (2012). Comparison of allogeneic vs autologous bone marrow-derived mesenchymal stem cells delivered by transendocardial injection in patients with ischemic cardiomyopathy: the POSEIDON randomized trial. JAMA.

[61] Packham D.K., Fraser I.R., Kerr P.G., Segal K.R. (2016). Allogeneic mesenchymal precursor cells (MPC) in diabetic nephropathy: a randomized, placebo-controlled, dose escalation study. EBio Med.

[62] Barnhoorn M.C., Wasser M., Roelofs H. (2020). Long-term evaluation of allogeneic bone marrow-derived mesenchymal stromal cell therapy for crohn's disease perianal fistulas. J Crohns Colitis.

[63] García-Sancho J., Sánchez A., Vega A., Noriega D.C., Nocito M. (2017). Influence of HLA matching on the efficacy of allogeneic mesenchymal stromal cell therapies for osteoarthritis and degenerative disc disease. Transplant Direct.

[64] Rowan K.M., Kerr J.H., Major E., McPherson K., Short A., Vessey M.P. (1993). Intensive care society's APACHE II study in Britain and Ireland–II: outcome comparisons of intensive care units after adjustment for case mix by the American APACHE II method. BMJ.

[65] Simonson O.E., Ståhle E., Hansen T. (2020). Five-year follow-up after mesenchymal stromal cell–based treatment of severe acute respiratory distress syndrome. Am J Respir Crit Care Med.

[66] Gorman E., Shankar-Hari M., Hopkins P. (2020). Repair of acute respiratory distress syndrome by stromal cell administration in COVID-19 (REALIST-COVID-19): a structured summary of a study protocol for a randomised. Trials.

