# Supplementary material for: Repair of acute respiratory distress syndrome by stromal cell administration (REALIST) trial: A phase 1 trial
Source: eClinicalMedicine. 2021 Oct 24;41:101167. doi: 10.1016/j.eclinm.2021.101167 (PMC8551601; doi:10.1016/j.eclinm.2021.101167)
Supplement: Supplementary file 1 [file mmc1.docx]

| **Supplemental Table 1: Primary and secondary outcome variables** | | | | | | |
| --- | --- | --- | --- | --- | --- | --- |
|  | | | **100 x 10^6^**  **n=3** | **200 x 10^6^**  **n=3** | **400 x 10^6^**  **n=3** | **Total**  **n=9** |
| **Primary safety outcome; incidence of SAEs** | | | | | | |
| **Number of events *** | | | 2(50·0%) | 1(25·0%) | 1(25·0%) | 4(100·0%) |
| **Number of patients †** | | | 1(33·3%) | 1(33·3%) | 1(33·3%) | 3(33·3%) |
| **Secondary Outcomes** | | | | | | |
| **Oxygenation Index (cmH_2_O/kPa)** | **Baseline** | **Observed Values** | n=2  44·4(16·1) | 133·7(141·0) | 62·2(21·1) | n=8  84·6(86·9) |
|  | **Day 4** | **Observed Values** | n=1  66·7 | n=1  195·8 | n=2  117·6 (26·5) | n=4  124·4(55·5) |
|  |  | **Imputed Values‡** | n=2  47·0(27·9) | 107·5(76·5) | 94·0(44·9) | n=8  87·3(55·0) |
|  | **Day 7** | **Observed Values** | n=0 | n=1  79.4 | n=2  74.8(1.3) | n=3  76.3(2.8) |
|  |  | **Imputed Values‡** | n=2  47·0(27·9) | 68·7(9·6) | 61·5(23·1) | n=8  60·6(19·3) |
|  | **Day 14** | **Observed Values** | n=0 | n=1  106.2 | n=1  79.1 | n=2  92.7(19.1) |
|  |  | **Imputed Values‡** | n=2  36·0(12·3) | 78·7(23·8) | 65·2(26·4) | n=8  63·0(26·5) |
| **P/F Ratio (PaO_2_/FiO_2_)** | **Baseline** | **Observed Values** | 21·7(8·2) | 12·7(4·9) | 21·2(3·6) | 18·5(6·7) |
|  | **Day 4** | **Observed Values** | 33·8(9·2) | 27·0(11·0) | 20·5(14·4) | 27·1(11·7) |
|  |  | **Imputed Values‡** | 33·8(9·2) | 27·0(11·0) | 20·5(14·4) | 27·1(11·7) |
|  | **Day 7** | **Observed Values** | 28·8(10·0) | 25·9(1·7) | 26·2(6·0) | 27·0(6·1) |
|  |  | **Imputed Values‡** | 28·8(10·0) | 25·9(1·7) | 26·2(6·0) | 27·0(6·1) |
|  | **Day 14** | **Observed Values** | n=1  32·3 | n=1  21·7 | n=1  20·2 | n=3  24·7(6·6) |
|  |  | **Imputed Values‡** | 32·1(0·6) | 23·6(3·2) | 29·4(14·5) | 28·4(8·3) |
| **Respiratory Compliance (ml/cmH_2_O)** | **Baseline** | **Observed Values** | n=2  41·4(9·6) | 25·0(5·1) | 28·4(10·2) | n=8  30·4(9·9) |
|  | **Day 4** | **Observed Values** | n=1  20·0 | n=0 | n=2  44·0 (18·0) | n=3  36·0(18·8) |
|  |  | **Imputed Values‡** | n=2  31·1(15·6) | 27·6(5·7) | 36·8(17·7) | n=8  31·9(12·4) |
|  | **Day 7** | **Observed Values** | n=0 | n=0 | n=2  21.5(2.3) | n=2  21.5(2.3) |
|  |  | **Imputed Values‡** | n=2  31·1(15·6) | 27·6(5·7) | 26·3(8·6) | n=8  28·0(8·3) |
|  | **Day 14** | **Observed Values** | n=0 | n=0 | n=1  20.4 | n=1  20.4 |
|  |  | **Imputed Values‡** | n=2  35·2(9·8) | 30·2(8·2) | 25·6(9·0) | n=8  29·8(8·5) |
| **Driving Pressure (cmH_2_O)** | **Baseline** | **Observed Values** | n=2  9·0(1·4) | 18·0(2·6) | 16·7(4·5) | n=8  15·3(4·8) |
|  | **Day 4** | **Observed Values** | n=1  20·0 | n=0 | n=2  13·0(4·2) | n=3  15·3(5·0) |
|  |  | **Imputed Values‡** | n=2  14·5(7·8) | 16·7(4·2) | 15·3(5·0) | n=8  15·6(4·7) |
|  | **Day 7** | **Observed Values** | n=0 | n=0 | n=2  18·0(2·8) | n=2  18·0(2·8) |
|  |  | **Imputed Values‡** | n=2  14·5(7·8) | 16·7(4·2) | 17·0(2·6) | n=8  16·3(4·1) |
|  | **Day 14** | **Observed Values** | n=0 | n=0 | n=1  18·0 | n=1  18·0 |
|  |  | **Imputed Values‡** | n=2  11·0(2·8) | 15·3(2·5) | 17·7(2·5) | n=8  15·1(3·5) |
| **Sequential Organ Failure Assessment (SOFA) score** | **Baseline** | **Observed Values** | n=2  12·0(4·2) | 16·3(4·6) | 10·3(3·2) | n=8  13·0(4·4) |
|  | **Day 4** | **Observed Values** | 5·7(6·0) | 13·0(5·0) | 7·0(3·6) | 8·6(5·5) |
|  |  | **Imputed Values‡** | 5·7(6·0) | 13·0(5·0) | 7·0(3·6) | 8·6(5·5) |
|  | **Day 7** | **Observed Values** | n=2  5·5(6·4) | 15·3(4·5) | 7·3(7·5) | n=8  9·9(7·0) |
|  |  | **Imputed Values‡** | 4·0(5·2) | 15·3(4·5) | 7·3(7·5) | 8·9(7·2) |
|  | **Day 14** | **Observed Values** | n=1  4·0 | n=1  21·0 | n=1  4·0 | n=3  9·7(9·8) |
|  |  | **Imputed Values‡** | 2·0(2·0) | 17·3(3·2) | 6·7(8·3) | 8·7(8·2) |
| Mean (SD), median[IQR] or n(%) presented  *Row percentages displayed  †Percentages calculated based on total number of patients recruited to each group  ‡Imputed Values based on last value carried forward | | | | | | |
