# Supplementary material for: Repair of acute respiratory distress syndrome by stromal cell administration (REALIST) trial: A phase 1 trial
Source: eClinicalMedicine. 2021 Oct 24;41:101167. doi: 10.1016/j.eclinm.2021.101167 (PMC8551601; doi:10.1016/j.eclinm.2021.101167)
Supplement: Supplementary file 2 [file mmc2.docx]

| **Supplemental Table 2: Biomarker studies at day 0, 4, 7 and 14** | | | | | | | |
| --- | --- | --- | --- | --- | --- | --- | --- |
| Patient | Timepoint | IL6 (pg/ml) | IL8 (pg/ml) | IL18 (ng/ml) | Ang2 (ng/ml) | ICAM-1 (ng/ml) | SP-D (ng/ml) |
| **100 x 10^6^ dose cohort** | | | | | | | |
| 1 | Baseline | 600* | 1022·80 | 3·75* | 59·70 | 330·72 | 1·56 |
|  | Day 4 | 337·55 | 256·00 | 3·58 | 9·94 | 328·56 | 31·06 |
|  | Day 7 | 514·4 | 104·35 | 1·02 | 13·80 | 312·83 | 18·70 |
|  | Day 14 | 131·04 | 55·99 | 1·74 | 12·38 | 350·56 | 19·06 |
| 2 | Baseline | 81·29 | 31·3** | 1·60 | 6·98 | 277·44 | 19·41 |
|  | Day 4 | 201·80 | 41·32 | 2·30 | 11·40 | 258·59 | 14·71 |
|  | Day 7 | 48·95 | 42·62 | 1·86 | 5·11 | 272·96 | 16·20 |
|  | Day 14 | 35·41 | 34·66 | 1·82 | 4·65 | 262·14 | 21·24 |
| 3 | Baseline | 26·92 | 31·3** | 2·91 | 6·76 | 191·85 | 3·30 |
|  | Day 4 | 49·43 | 31·3** | 2·52 | 2·54 | 253·76 | 3·71 |
|  | Day 7 | 139·22 | 32·65 | 2·71 | 4·74 | 313·09 | 4·95 |
|  | Day 14 | - | - | - | - | - | - |
| **200 x 10^6^ dose cohort** | | | | | | | |
| 4 | Baseline | 66·90 | 151·99 | 3·75 | 14·23 | 599·29 | 14·79 |
|  | Day 4 | 13·47 | 31·83 | 3·75 | 2·36 | 653·77 | 48·85 |
|  | Day 7 | - | - | - | - | - | - |
|  | Day 14 | - | - | - | - | - | - |
| 5 | Baseline | 600* | 686·02 | 2·38 | 16·47 | 319·26 | 5·64 |
|  | Day 4 | - | - | - | - | - | - |
|  | Day 7 | 600* | 443·53 | 3·27 | 27·85 | 822·83 | 8·85 |
|  | Day 14 | - | - | - | - | - | - |
| 6 | Baseline | 295·65 | 75·29 | 0·99 | 10·89 | 335·30 | 23·90 |
|  | Day 4 | 51·49 | 58·35 | 1·03 | 10·22 | 388·53 | 10·88 |
|  | Day 7 | 83·42 | 37·22 | 1·06 | 11·54 | 282·35 | 11·40 |
|  | Day 14 | - | - | - | - | - | - |
| **400 x 10^6^ dose cohort** | | | | | | | |
| 7 | Baseline | 417·26 | 126·61 | 1·39 | 44·99 | 338·66 | 11·82 |
|  | Day 4 | 89·95 | 95·01 | 1·85 | 16·22 | 331·27 | 21·62 |
|  | Day 7 | 91·71 | 46·65 | 1·84 | 12·60 | 391·24 | 14·15 |
|  | Day 14 | - | - | - | - | - | - |
| 8 | Baseline | 600* | 202·55 | 1·91 | 17·71 | 357·03 | 7·01 |
|  | Day 4 | 600* | 115·40 | 3·25 | 16·27 | 420·25 | 14·31 |
|  | Day 7 | 49·29 | 31·3** | 2·46 | 4·19 | 394·71 | 18·20 |
|  | Day 14 | 35·17 | 31·3** | 3·20 | 5·52 | 222·26 | 15·55 |
| 9 | Baseline | 259·38 | 31·3** | 0·44 | 3·10 | 173·71 | 9·59 |
|  | Day 4 | 277·97 | 31·3** | 0·59 | 1·96 | 435·06 | 14·45 |
|  | Day 7 | 49·28 | 31·3** | 0·68 | 1·70 | 323·29 | 13·26 |
|  | Day 14 | 20·35 | 31·3** | 0·57 | 1·32 | 159·54 | 5·98 |
| *measurement was above upper limit of assay  ** measurement was below lower limit of assay | | | | | | | |
