# Supplementary material for: Repair of acute respiratory distress syndrome by stromal cell administration (REALIST) trial: A phase 1 trial
Source: eClinicalMedicine. 2021 Oct 24;41:101167. doi: 10.1016/j.eclinm.2021.101167 (PMC8551601; doi:10.1016/j.eclinm.2021.101167)
Supplement: Supplementary file 3 [file mmc3.docx]

| **Supplemental Table 3: Routine clinical laboratory measurements daily to day 14** | | | | |  |
| --- | --- | --- | --- | --- | --- |
|  | **Dose 1**  **n = 3** | **Dose 2**  **n = 3** | **Dose 3**  **n = 3** | **Total**  **n=9** |  |
| **AST (U/L)** | | | | | |
| **Day 0** | n=3  103.7(157.1) | n=2  232.5(266.6) | n=3  136.7(21.4) | n=8  148.3(142.4) |  |
| **Day 1** | n=2  14.0(1.4) | n=2  234.0(267.3) | n=3  861.7(1299.3) | n=7  440.1(859.2) |  |
| **Day 2** | n=2  263.5(355.7) | n=2  264.5(300.5) | n=3  751.0(1144.0) | n=7  472.7(735.0) |  |
| **Day 3** | n=2  126(149.9) | n=1  76.0 | n=3  346.7(475.7) | n=6  228.0(335.1) |  |
| **Day 4** | n=3  74.3(83.6) | n=2  152.0(101.8) | n=3  184(156.9) | n=8  134.9(114.9) |  |
| **Day 5** | n=3  70.0(80.4) | n=2  134.5(55.9) | n=2  108.0(48.1) | n=7  99.3(62.7) |  |
| **Day 6** | n=2  54.5(57.3) | n=2  110.5(58.7) | n=3  72.7(25.9) | n=7  78.3(43.5) |  |
| **Day 7** | n=2  38.0(32.5) | n=2  117.0(72.1) | n=3  110.0(84.6) | n=7  91.4(69.1) |  |
| **Day 8** | n=3  30.7(22.7) | n=1  60.0 | n=2  85.5(72.8) | n=6  53.8(44.7) |  |
| **Day 9** | n=2  30.0(24.0) | n=1  97.0 | n=2  63.5(26.2) | n=5  56.8(33.2) |  |
| **Day 10** | n=1  41.0 | n=0 | n=2  54.5(10.6) | n=3  50.0(10.8) |  |
| **Day 11** | n=1  36.0 | n=0 | n=1  48.0 | n=2  42.0(8.5) |  |
| **Day 12** | n=1  44.0 | n=0 | n=1  136.0 | n=2  90.0(65.1) |  |
| **Day 13** | n=1  38.0 | n=0 | n=0 | n=1  38.0 |  |
| **Day 14** | n=1  55.0 | n=0 | n=1  181.0 | n=2  118.0(89.1) |  |
| **ALT (U/L)** | | | | | |
| **Day 0** | n=3  19.3(16.3) | n=3  72.7(45.4) | n=3  74.7(59.5) | n=9  55.6(47.0) |  |
| **Day 1** | n=2  11.0(2.8) | n=3  70.3(43.3) | n=3  383.3(598.2) | n=8  172.9(365.7) |  |
| **Day 2** | n=2  43.0(49.5) | n=3  81.0(49.2) | n=3  454.0(724.1) | n=8  211.4(437.5) |  |
| **Day 3** | n=2  48.5(54.4) | n=2  83.5(72.8) | n=3  345.3(535.8) | n=7  185.7(345.8) |  |
| **Day 4** | n=3  40.7(47.3) | n=3  115.7(79.0) | n=3  261.0(350.8) | n=9  139.1(205.7) |  |
| **Day 5** | n=3  55.7(73.2) | n=3  124.0(77.5) | n=2  242.0(261.6) | n=8  127.9(137.8) |  |
| **Day 6** | n=2  63.5(77.1) | n=3  114.0(64.1) | n=3  121.7(115.7) | n=8  104.3(80.6) |  |
| **Day 7** | n=2  51.0(62.2) | n=3  110.7(53.4) | n=3  113.3(64.4) | n=8  96.8(57.9) |  |
| **Day 8** | n=3  37.7(37.6) | n=2  99.0(62.2) | n=2  80.0(63.6) | n=7  67.3(51.2) |  |
| **Day 9** | n=2  39.5(41.7) | n=2  95.5(75.7) | n=2  71.5(46.0) | n=6  68.8(50.5) |  |
| **Day 10** | n=1  55.0 | n=1  136.0 | n=2  63.5(33.2) | n=4  79.5(42.5) |  |
| **Day 11** | n=1  51.0 | n=1  114.0 | n=1  44.0 | n=3  69.7(38.6) |  |
| **Day 12** | n=1  47.0 | n=1  107.0 | n=1  109.0 | n=3  87.7(35.2) |  |
| **Day 13** | n=1  38.0 | n=1  103.0 | n=0 | n=2  70.5(46.0) |  |
| **Day 14** | n=1  46.0 | n=1  145.0 | n=1  188.0 | n=3  126.3(72.8) |  |
| **ALP (U/L)** | | | | | |
| **Day 0** | n=3  51.3(30.0) | n=3  97.0(70.0) | n=3  52.3(14.2) | n=9  66.9(44.8) |  |
| **Day 1** | n=2  76.0(1.4) | n=3  114.7(100.9) | n=3  55.0(14.0) | n=8  82.6(61.2) |  |
| **Day 2** | n=3  66.0(18.0) | n=3  157.7(165.4) | n=3  67.7(21.1) | n=9  97.1(95.4) |  |
| **Day 3** | n=3  85.0(7.0) | n=3  279.7(343.5) | n=3  120.7(68.5) | n=9  161.8(196.8) |  |
| **Day 4** | n=3  86.3(7.1) | n=3  238.0(246.0) | n=3  148.7(80.1) | n=9  157.7(145.3) |  |
| **Day 5** | n=3  85.3(10.1) | n=3  230.0(212.8) | n=2  144.0(87.7) | n=8  154.3(136.3) |  |
| **Day 6** | n=3  87.3(14.8) | n=3  261.0(269.6) | n=3  148.0(54.1) | n=9  165.4(157.4) |  |
| **Day 7** | n=3  87.0(20.7) | n=3  319.3(361.2) | n=3  165.0(60.6) | n=9  190.4(210.1) |  |
| **Day 8** | n=3  92.7(22.0) | n=2  111.0(58.0) | n=2  189.5(12.0) | n=7  125.6(52.2 ) |  |
| **Day 9** | n=3  102.7(18.1) | n=2  112.0(79.2) | n=2  201.5(17.7) | n=7  133.6(58.1) |  |
| **Day 10** | n=1  130.0 | n=1  155.0 | n=2  167.0(9.9) | n=4  154.8(18.4) |  |
| **Day 11** | n=1  159.0 | n=1  155.0 | n=1  165.0 | n=3  159.7(5.0) |  |
| **Day 12** | n=1  145.0 | n=1  171.0 | n=1  221.0 | n=3  179.0(38.6) |  |
| **Day 13** | n=1  126.0 | n=1  177.0 | n=0 | n=2  151.5(36.1) |  |
| **Day 14** | n=1  110.0 | n=1  185.0 | n=1  211.0 | n=3  168.7(52.4) |  |
| **CRP (mg/L)** | | | | | |
| **Day 0** | n=3  238.8(136.1) | n=2  199.1(136.3) | n=3  472.6(188.4) | n=8  316.5(187.2) |  |
| **Day 1** | n=2  170.2(148.6) | n=3  178.3(58.1) | n=3  449.4(126.1) | n=8  277.9(169.8) |  |
| **Day 2** | n=3  244.3(180.3) | n=3  175.9(18.7) | n=3  415.6(74.5) | n=9  278.6(145.0) |  |
| **Day 3** | n=2  148.4(110.4) | n=3  158.0(118.5) | n=3  355.3(82.2) | n=8  229.6(136.1) |  |
| **Day 4** | n=2  139.1(25.1) | n=3  146.9(161.5) | n=3  374.8(152.2) | n=8  230.4(168.7) |  |
| **Day 5** | n=3  198.1(126.7) | n=3  134.6(171.8) | n=2  312.2(233.3) | n=8  202.8(161.9) |  |
| **Day 6** | n=2  207.6(134.8) | n=3  116.2(155.7) | n=3  290.9(180.9) | n=8  204.6(159.4) |  |
| **Day 7** | n=2  245.5(226.9) | n=3  115.6(160.6) | n=3  279.2(113.1) | n=8  209.4(156.9) |  |
| **Day 8** | n=2  230.7(194.0) | n=2  152.3(156.6) | n=2  180.0(17.4) | n=6  187.6(117.3) |  |
| **Day 9** | n=2  202.5(194.0) | n=1  251.0 | n=2  178.9(111.9) | n=5  202.8(115.8) |  |
| **Day 10** | n=1  316.4 | n=1  193.0 | n=2  186.9(195.7) | n=4  220.8(129.8) |  |
| **Day 11** | n=1  281.7 | n=1  164.0 | n=1  333.4 | n=3  259.7(86.8) |  |
| **Day 12** | n=1  270.1 | n=1  155.0 | n=1  308.5 | n=3  244.5(79.9) |  |
| **Day 13** | n=1  300.5 | n=1  156.0 | n=1  278.6 | n=3  245.0(77.9) |  |
| **Day 14** | n=1  264.2 | n=1  136.0 | n=0 | n=2  200.1(90.7) |  |
| **PT (s)** | | | | | |
| **Day 0** | n=3  10.7(0.9) | n=3  13.0(1.4) | n=3  12.0(0.1) | n=9  11.9(1.3) |  |
| **Day 1** | n=3  10.4(0.2) | n=3  12.5(0.9) | n=3  11.3(0.4) | n=9  11.4(1.0) |  |
| **Day 2** | n=3  10.3(0.3) | n=2  11.1(1.7) | n=3  11.0(0.3) | n=8  10.8(0.8) |  |
| **Day 3** | n=3  10.6(0.5) | n=2  11.0(1.7) | n=3  10.9(0.7) | n=8  10.8(0.8) |  |
| **Day 4** | n=2  10.4(0.6) | n=2  11.8(1.6) | n=2  10.9(0.5) | n=6  11.0(1.0) |  |
| **Day 5** | n=3  10.7(0.2) | n=2  12.0(1.5) | n=2  10.9(0.8) | n=7  11.1(0.9) |  |
| **Day 6** | n=2  10.6(0.1) | n=2  11.9(1.8) | n=3  10.9(0.5) | n=7  11.1(0.9) |  |
| **Day 7** | n=2  10.9(0.0) | n=1  12.8 | n=2  11.0(0.9) | n=5  11.3(1.0) |  |
| **Day 8** | n=2  11.1(0.1) | n=0 | n=2  11.3(0.2) | n=4  11.2(0.2) |  |
| **Day 9** | n=2  10.8(0.1) | n=1  16.7 | n=2  11.4(0.3) | n=5  12.2(2.5) |  |
| **Day 10** | n=1  11.4 | n=0 | n=2  11.6(0.4) | n=3  11.5(0.3) |  |
| **Day 11** | n=1  11.2 | n=0 | n=1  11.3 | n=2  11.3(0.1) |  |
| **Day 12** | n=1  11.1 | n=0 | n=1  10.9 | n=2  11.0(0.1) |  |
| **Day 13** | n=1  10.9 | n=0 | n=1  10.9 | n=2  10.9(0.0) |  |
| **Day 14** | n=1  11.3 | n=0 | n=0 | n=1  11.3 |  |
| **APTT (s)** | | | | | |
| **Day 0** | n=3  38.2(18.6) | n=3  40.5(8.4) | n=1  27.2 | n=7  37.6(12.7) |  |
| **Day 1** | n=2  26.3(1.0) | n=3  38.2(5.2) | n=1  28.3 | n=6  32.6(7.0) |  |
| **Day 2** | n=2  27.8(4.2) | n=2  36.1(0.2) | n=2  41.8(8.8) | n=6  35.2(7.7) |  |
| **Day 3** | n=3  26.9(1.7) | n=2  31.9(6.1) | n=3  30.6(3.9) | n=8  29.5(3.9) |  |
| **Day 4** | n=2  27.7(4.2) | n=2  31.4(2.7) | n=2  30.0(2.6) | n=6  29.7(3.0) |  |
| **Day 5** | n=3  26.6(1.6) | n=2  31.5(6.0) | n=2  28.4(1.7) | n=7  28.5(3.5) |  |
| **Day 6** | n=1  27.7 | n=2  31.1(7.4) | n=2  31.0(3.8) | n=5  30.4(4.4) |  |
| **Day 7** | n=2  28.9(0.4) | n=1  33.0 | n=2  29.0(6.9) | n=5  29.7(3.9) |  |
| **Day 8** | n=2  28.4(0.7) | n=0 | n=2  22.1(0.6) | n=4  25.3(3.7) |  |
| **Day 9** | n=2  27.7(0.6) | n=1  46.0 | n=2  21.1(1.6) | n=5  28.7(10.2) |  |
| **Day 10** | n=1  29.5 | n=0 | n=2  21.4(2.0) | n=3  24.1(4.9) |  |
| **Day 11** | n=1  29.9 | n=0 | n=1  22.1 | n=2  26.0(5.5) |  |
| **Day 12** | n=1  30.3 | n=0 | n=1  21.9 | n=2  26.1(5.9) |  |
| **Day 13** | n=1  31.0 | n=0 | n=1  22.9 | n=2  27.0(5.7) |  |
| **Day 14** | n=1  30.8 | n=0 | n=0 | n=1  30.8 |  |
| **Fibrinogen (g/l)** | | | | | |
| **Day 0** | n=3  4.6(1.2) | n=3  3.5(0.5) | n=3  5.3(0.9) | n=9  4.4(1.1) |  |
| **Day 1** | n=3  4.8(1.4) | n=3  3.3(0.6) | n=3  5.4(0.5) | n=9  4.5(1.2) |  |
| **Day 2** | n=3  4.6(1.7) | n=2  3.2(0.8) | n=3  5.6(0.2) | n=8  4.6(1.4) |  |
| **Day 3** | n=3  4.7(1.1) | n=2  2.7(1.6) | n=3  5.9(0.2) | n=8  4.7(1.6) |  |
| **Day 4** | n=2  4.7(0.1) | n=2  2.5(1.6) | n=2  5.9(0.0) | n=6  4.4(1.7) |  |
| **Day 5** | n=3  5.1(0.6) | n=2  2.2(1.3) | n=2  6.0(0.1) | n=7  4.5(1.7) |  |
| **Day 6** | n=2  5.4(0.9) | n=2  2.0(0.8) | n=3  5.9(0.2) | n=7  4.6(1.9) |  |
| **Day 7** | n=2  5.6(0.8) | n=2  4.1(2.2) | n=2  6.3(0.2) | n=6  5.3(1.5) |  |
| **Day 8** | n=2  5.6(0.6) | n=1  6.4 | n=2  6.0(0.2) | n=5  5.9(0.5) |  |
| **Day 9** | n=2  5.7(0.5) | n=2  4.5(2.9) | n=2  6.0(0.1) | n=6  5.4(1.5) |  |
| **Day 10** | n=1  6.0 | n=1  4.7 | n=2  5.8(0.2) | n=4  5.6(0.6) |  |
| **Day 11** | n=1  6.0 | n=1  4.7 | n=1  5.9 | n=3  5.5(0.7) |  |
| **Day 12** | n=1  6.0 | n=1  4.2 | n=1  5.9 | n=3  5.4(1.0) |  |
| **Day 13** | n=1  5.8 | n=1  4.2 | n=1  6.1 | n=3  5.4(1.0) |  |
| **Day 14** | n=1  5.6 | n=1  2.9 | n=0 | n=2  4.3(1.9) |  |
| **Hb (g/l)** | | | | | |
| **Day 0** | n=3  95.7(19.5) | n=3  104.7(22.1) | n=3  103.7(17.0) | n=9  101.3(17.5) |  |
| **Day 1** | n=3  103.7(18.2) | n=2  106.5(27.6) | n=3  102(15.6) | n=8  103.8(16.6) |  |
| **Day 2** | n=3  97.7(16.3) | n=3  99.3(15.5) | n=3  99.3(11.4) | n=9  98.8(12.6) |  |
| **Day 3** | n=3  98.3(10.5) | n=3  101.3(18.8) | n=3  99.7(9.0) | n=9  99.8(11.7) |  |
| **Day 4** | n=3  98.7(12.3) | n=3  98.0(18.2) | n=3  100.7(11.4) | n=9  99.1(12.5) |  |
| **Day 5** | n=3  94.7(11.0) | n=3  89.0(14.8) | n=3  96.0(8.9) | n=9  93.2(10.7) |  |
| **Day 6** | n=3  89.3(2.1) | n=3  92.0(19.7) | n=3  92.0(8.9) | n=9  91.1(10.9) |  |
| **Day 7** | n=3  84.0(2.6) | n=3  93.7(10.6) | n=3  99.0(7.8) | n=9  92.2(9.4) |  |
| **Day 8** | n=3  83.7(2.3) | n=2  94.5(17.7) | n=3  90.3(6.7) | n=8  88.9(9.0) |  |
| **Day 9** | n=3  84.3(4.7) | n=2  88.0(24.0) | n=2  95.0(21.2) | n=7  88.4(14.2) |  |
| **Day 10** | n=1  81.0 | n=1  93.0 | n=2  91.5(21.9) | n=4  89.3(13.8) |  |
| **Day 11** | n=1  82.0 | n=1  95.0 | n=2  87.5(23.3) | n=4  88.0(14.5) |  |
| **Day 12** | n=1  73.0 | n=1  90.0 | n=1  73.0 | n=3  78.7(9.8) |  |
| **Day 13** | n=1  73.0 | n=1  90.0 | n=1  69.0 | n=3  77.3(11.2) |  |
| **Day 14** | n=1  61.0 | n=1  76.0 | n=1  78.0 | n=3  71.7(9.3) |  |
| **WBC (x10^9^/L)** | | | | | |
| **Day 0** | n=3  8.4(4.8) | n=3  13.2(4.5) | n=3  9.0(4.0) | n=9  10.2(4.5) |  |
| **Day 1** | n=3  12.3(3.3) | n=2  15.0(3.6) | n=3  7.5(4.4) | n=8  11.2(4.6) |  |
| **Day 2** | n=3  10.1(1.2) | n=3  11.5(2.7) | n=3  7.9(3.6) | n=9  9.8(2.8) |  |
| **Day 3** | n=3  13.7(2.2) | n=3  13.7(5.0) | n=3  6.2(2.8) | n=9  11.2(4.8) |  |
| **Day 4** | n=3  14.3(3.5) | n=3  14.6(8.0) | n=3  9.0(4.4) | n=9  12.6(5.6) |  |
| **Day 5** | n=3  13.5(2.6) | n=3  14.7(8.4) | n=3  11.2(6.5) | n=9  13.1(5.7) |  |
| **Day 6** | n=3  13.1(1.6) | n=3  13.6(8.2) | n=3  11.0(3.9) | n=9  12.6(4.8) |  |
| **Day 7** | n=3  13.4(1.6) | n=3  20.0(13.6) | n=3  15.9(5.6) | n=9  16.4(7.9) |  |
| **Day 8** | n=3  13.5(5.5) | n=2  47.7(0.4) | n=3  11.9(1.9) | n=8  21.5(16.5) |  |
| **Day 9** | n=3  14.9(5.7) | n=2  55.6(1.0) | n=2  14.4(3.4) | n=7  26.4(20.3) |  |
| **Day 10** | n=1  21.9 | n=1  49.5 | n=2  12.1(2.1) | n=4  23.9(17.7) |  |
| **Day 11** | n=1  18.5 | n=1  48.0 | n=2  9.9(2.6) | n=4  21.6(18.2) |  |
| **Day 12** | n=1  15.3 | n=1  47.1 | n=1  7.9 | n=3  23.4(20.8) |  |
| **Day 13** | n=1  15.5 | n=1  35.8 | n=1  7.3 | n=3  19.5(14.7) |  |
| **Day 14** | n=1  11.8 | n=1  36.9 | n=1  7.1 | n=3  18.6(16.0) |  |
| **Neutrophils (x10^9^/L)** | | | | | |
| **Day 0** | n=3  6.6(4.1) | n=3  10.8(4.5) | n=3  8.1(3.4) | n=9  8.5(4.0) |  |
| **Day 1** | n=3  8.8(0.9) | n=2  12.2(4.7) | n=3  6.5(3.6) | n=8  8.8(3.5) |  |
| **Day 2** | n=3  8.3(1.1) | n=3  9.6(2.8) | n=3  6.6(2.7) | n=9  8.2(2.4) |  |
| **Day 3** | n=3  11.6(1.9) | n=3  12.0(4.4) | n=3  5.1(2.8) | n=9  9.6(4.4) |  |
| **Day 4** | n=3  12.1(2.5) | n=2  16.0(5.7) | n=3  7.6(4.4) | n=8  11.4(4.9) |  |
| **Day 5** | n=3  11.2(1.6) | n=3  11.5(6.8) | n=3  8.7(6.4) | n=9  10.5(4.9) |  |
| **Day 6** | n=3  11.2(1.0) | n=3  10.4(6.3) | n=3  8.5(4.5) | n=9  10.0(4.1) |  |
| **Day 7** | n=3  11.4(1.7) | n=3  16.5(11.5) | n=3  13.7(6.1) | n=9  13.9(6.9) |  |
| **Day 8** | n=3  11.5(5.8) | n=2  36.8(9.1) | n=3  9.8(3.0) | n=8  17.2(13.1) |  |
| **Day 9** | n=1  12.8(5.9) | n=1  37.9 | n=2  12.3(2.7) | n=6  16.8(11.1) |  |
| **Day 10** | n=1  20.3 | n=1  41.6 | n=2  9.8(1.1) | n=4  20.4(15.0) |  |
| **Day 11** | n=1  16.3 | n=1  39.8 | n=2  7.7(1.3) | n=4  17.9(15.2) |  |
| **Day 12** | n=1  13.1 | n=1  38.6 | n=1  6.4 | n=3  19.4(17.0) |  |
| **Day 13** | n=1  13.2 | n=1  28.6 | n=1  5.4 | n=3  15.7(11.8) |  |
| **Day 14** | n=1  9.8 | n=1  26.8 | n=1  4.8 | n=3  13.8(11.5) |  |
| **Lowest eGFR (mL/min)** | | | | | |
| **Day 0** | n=3  45.3(15.8) | n=3  45.3(13.3) | n=3  48.3(10.7) | n=9  46.3(11.7) |  |
| **Day 1** | n=3  43.0(12.2) | n=3  46.0(13.1) | n=3  49.7(10.5) | n=9  46.2(10.8) |  |
| **Day 2** | n=3  51.0(8.2) | n=3  43.0(18.7) | n=3  53.0(12.1) | n=9  49.0(12.7) |  |
| **Day 3** | n=3  37.7(20.7) | n=3  48.7(15.5) | n=3  54.3(9.8) | n=9  46.9(15.7) |  |
| **Day 4** | n=3  40.3(17.1) | n=3  55.3(22.2) | n=3  57.7(4.0) | n=9  51.1(16.3) |  |
| **Day 5** | n=3  44.3(14.6) | n=3  49.3(9.7) | n=3  52.3(13.3) | n=9  48.7(11.6) |  |
| **Day 6** | n=3  50.7(8.3) | n=3  47.0(13.0) | n=3  55.7(7.5) | n=9  51.1(9.4) |  |
| **Day 7** | n=3  57.0(5.2) | n=3  40.3(17.1) | n=3  58.0(2.0) | n=9  51.8(12.4) |  |
| **Day 8** | n=3  55.7(7.5) | n=2  41.5(10.6) | n=3  58.7(2.3) | n=8  53.3(9.4) |  |
| **Day 9** | n=3  58.0(3.5) | n=2  30.0(8.5) | n=2  60.0(0.0) | n=7  50.6(14.6) |  |
| **Day 10** | n=1  56.0 | n=1  35.0 | n=2  60.0(0.0) | n=4  52.8(12.0) |  |
| **Day 11** | n=1  59.0 | n=1  40.0 | n=2  60.0(0.0) | n=4  54.8(9.8) |  |
| **Day 12** | n=1  54.0 | n=1  50.0 | n=1  60.0 | n=3  54.7(5.0) |  |
| **Day 13** | n=1  58.0 | n=1  49.0 | n=1  60.0 | n=3  55.7(5.9) |  |
| **Day 14** | n=1  60.0 | n=1  66.0 | n=1  60.0 | n=3  62.0(3.5) |  |
| **Highest Urea (mmol/L)** | | | | | |
| **Day 0** | n=3  12.6(6.3) | n=3  10.4(5.2) | n=3  8.8(2.7) | n=9  10.6(4.6) |  |
| **Day 1** | n=3  13.7(5.8) | n=3  9.6(5.4) | n=3  8.6(3.3) | n=9  10.7(4.9) |  |
| **Day 2** | n=3  14.2(3.9) | n=3  9.9(4.4) | n=3  8.5(4.8) | n=9  10.9(4.6) |  |
| **Day 3** | n=3  13.1(5.6) | n=3  9.9(6.7) | n=3  8.5(5.3) | n=9  10.5(5.5) |  |
| **Day 4** | n=3  12.5(7.1) | n=3  9.0(4.6) | n=3  8.2(4.4) | n=9  9.9(5.1) |  |
| **Day 5** | n=3  12.7(8.5) | n=2  12.6(6.0) | n=3  10.4(5.6) | n=8  11.8(6.0) |  |
| **Day 6** | n=3  11.5(6.9) | n=3  13.4(6.3) | n=3  10.7(5.4) | n=9  11.9(5.5) |  |
| **Day 7** | n=3  11.2(5.3) | n=3  13.6(0.8) | n=3  11.4(6.2) | n=9  12.1(4.3) |  |
| **Day 8** | n=3  12.3(4.6) | n=2  16.9(2.5) | n=3  11.9(6.4) | n=8  13.3(4.8) |  |
| **Day 9** | n=3  11.6(3.5) | n=2  22.5(7.8) | n=2  13.4(3.5) | n=7  15.2(6.4) |  |
| **Day 10** | n=1  7.3 | n=1  19.7 | n=2  13.2(2.3) | n=4  13.4(5.2) |  |
| **Day 11** | n=1  8.3 | n=1  19.2 | n=2  12.7(2.8) | n=4  13.2(4.8) |  |
| **Day 12** | n=1  9.0 | n=1  18.1 | n=1  13.8 | n=3  13.6(4.6) |  |
| **Day 13** | n=1  9.4 | n=1  17.0 | n=1  15.7 | n=3  14.0(4.1) |  |
| **Day 14** | n=1  9.1 | n=1  10.2 | n=1  17.9 | n=3  12.4(4.8) |  |
| Mean (SD) presented | | | | |  |
