# Supplementary material for: Repair of acute respiratory distress syndrome by stromal cell administration (REALIST) trial: A phase 1 trial
Source: eClinicalMedicine. 2021 Oct 24;41:101167. doi: 10.1016/j.eclinm.2021.101167 (PMC8551601; doi:10.1016/j.eclinm.2021.101167)
Supplement: Supplementary file 4 [file mmc4.docx]

| Supplemental Table 4: Anti-HLA antibody response at day 0 and day 28 | | | | | | |
| --- | --- | --- | --- | --- | --- | --- |
| Patient | Dose cohort | Anti-HLA antibodies | | HLA IgG specificities detected | Common HLA antigens between ORBCEL-C and day 28 antibody response |  |
|  |  | Day 0 | Day 28 |  |  |  |
| 1 | 100 x10^6^ | Absent | Present | HLA-A*01, *23, *24, *25, *29, *34, *36, *66, *80 HLA- B*44, *45, *15:12, *15:16, *82 | HLA-A*01 |  |
| 2 | 100 x10^6^ | Absent | - |  |  |  |
| 3 | 100 x10^6^ | Absent | Absent |  |  |  |
| 4 | 200 x10^6^ | Absent | - |  |  |  |
| 5 | 200 x10^6^ | Absent | - |  |  |  |
| 6 | 200 x10^6^ | Absent | - |  |  |  |
| 7 | 400 x10^6^ | Absent | - |  |  |  |
| 8 | 400 x10^6^ | Absent | Present | HLA-*23, *24, *25, *32  HLA-B*13, *27, *37, *38, *44, *45, *47, *49, *51, *52, *53, *57, *58, *59, *15:12, *15:13, *15:16  HLA-C*17 | None |  |
| 9 | 400 x10^6^ | Absent | Absent |  |  |  |
