# Supplementary material for: Repair of acute respiratory distress syndrome by stromal cell administration (REALIST) trial: A phase 1 trial
Source: eClinicalMedicine. 2021 Oct 24;41:101167. doi: 10.1016/j.eclinm.2021.101167 (PMC8551601; doi:10.1016/j.eclinm.2021.101167)
Supplement: Supplementary file 6 [file mmc6.pdf]

REALIST
Phase 1

Repair of Acute Respiratory Distress Syndrome by Stromal Cell Administration

Protocol Number:	16154DMcA-AS
EudraCT Number:	2017-000584-33
Ethics Reference Number:	18/NE/0006
ClinicalTrials.gov	NCT03042143

STATISTICAL ANALYSIS PLAN

Final 1.0 08/07/2019

Northern Ireland Clinical Trials Unit
1st Floor Elliott Dynes
The Royal Hospitals
Grosvenor Rd
Belfast
BT12 6BA
Tel: +44 (0)28 9063 5794
Fax: +44 (0)28 9063 3328
Email: info@nictu.hscni.net

Contacts

Clíona McDowell – Head of Statistics
Christina Campbell – Biostatistician

This document and all preceding versions will be stored in the Trial Master File for this trial

Contents

STATISTICAL ANALYSIS PLAN 1

ABBREVIATIONS 4

1. Background and Design 6

2. Outcome measures 8

2.1 Primary outcome measure(s) 8
2.2 Secondary outcome measures 9
2.3 Exploratory Outcome Measures 9

3. Data 9

3.1 CRF Forms and variables 9
3.2 Management of datasets 10
3.3 Data completion schedule 10
3.4 Data verification 11
3.5 Data coding 11

4. Definition of terms 11

5. Sample Size Calculations 13

6. RANDOMISATION AND BLINDING 13

6.1 Randomisation 13
6.2 Blinding and Allocation Concealment 13

7. Analysis Principles 13

8. Analysis Details 15

8.1 Recruitment and follow-up patterns 15
8.2 CONSORT Flow Diagram – Dose 1 (100 x 10^6) 15
8.3 CONSORT Flow Diagram – Dose 2 (200 x 10^6) 16
8.4 CONSORT Flow Diagram – Dose 3 (400 x 10^6) 17
8.5 Safety 18
8.6 Baseline Characteristics 18
8.7 Trial treatment 18
8.8 Trial Outcomes 18
8.9 Main Clinical Lab Assessments 19

9. Additional Information 19

9.1 Trial Steering Committee (TSC) 19
9.2 Data Monitoring and Ethics Committee (DMEC) 19

10. Signatures of Approval 20

Appendix 1: Example Draft Summary Tables 21

ABBREVIATIONS

Abbreviation / Acronym	Full Wording
ABG	Arterial Blood Gas
AE	Adverse Event
ALI	Acute Lung Injury
APACHE	Acute Physiology and Chronic Health Evaluation
AR	Adverse Reaction
ARDS	Acute Respiratory Distress Syndrome
ATMP	Advanced Therapeutic Medicinal Product
BAL	Bronchoalveolar Lavage
BHSCT	Belfast Health and Social Care Trust
BM	Bone Marrow
CI	Chief Investigator
CFU-F	Colony Forming Unit Fibroblasts
CMP	Case Mix Programme
CO_2	Carbon Dioxide
CONSORT	Controlled Standards of Reporting Trials
CPAP	Continuous positive airway pressure
CRF	Case Report Form
Crs	Respiratory compliance
CRP	C-reactive protein
CTA	Clinical Trial Authorisation
CTIMP	Clinical Trial Investigational Medicinal Product
CTU	Clinical Trials Unit
CXR	Chest X-ray
DMEC	Data Monitoring and Ethics Committee
DLT	Dose Limiting Toxicity
DMP	Data Management Plan
DMSO	Dimethyl Sulfoxide
DNAR	Do Not Attempt Resuscitation
ECLS	Extracorporeal Life Support
ECMO	Extracorporeal Membrane Oxygenation
EKG	Electrocardiogram
ELISA	Enzyme-Linked Immunosorbent Assay
EudraCT	European Clinical Trials Database
FiO_2	Fraction of Inspired Oxygen
GP	General Practitioner
GCP	Good Clinical Practice
GMP	Good Manufacturing Practice
HLA Ab	Human Leukocyte Antigen Anti-bodies
HTA	Human Tissue Authority
IB	Investigator Brochure
ICH	International Conference on Harmonisation
ICNARC	Intensive Care National Audit & Research Centre
ICU	Intensive Care Unit
ISF	Investigator Site File

IV	Intravenous
LPS	Lipopolysaccharide
MDM	Monocyte-derived Macrophages
MHRA	Medicines and Healthcare products Regulatory Agency
MMP	Matrix metalloproteinases
MSC	Mesenchymal Stromal Cell
MTD	Maximal Tolerated Dose
NHS	National Health Service
NHSBT	National Health Service Blood and Transplant
NICTU	Northern Ireland Clinical Trials Unit
NETs	Neuroendocrine Tumors
NMBD	Neuromuscular Blocking Drugs
O_2	Oxygen
OI	Oxygenation Index
$PaCO_2$	Partial Pressure of Carbon Dioxide in arterial blood
PaO_2	Partial Pressure of Oxygen in arterial blood
PBW	Predicted Body Weight
PerLR	Personal Legal Representative
PEEP	Positive End Expiratory Pressure
P/F ratio	PaO_2/FiO_2 ratio
PI	Principal Investigator
PIS	Patient Information Sheet
ProfLR	Professional Legal Representative
QUB	Queens University Belfast
RAGE	Receptor for Advanced Glycation Endproducts
REC	Research Ethics Committee
RR	Respiratory Rate
SAE	Serious Adverse Event
SAP	Statistical Analysis Plan
SAPS	Simplified Acute Physiology Score
SDV	Source Data Verification
SOFA	Sequential Organ Failure Assessment
SOPs	Standard Operating Procedures
SP-D	Surfactant Protein-D
SUSAR	Suspected Unexpected Serious Adverse Reaction
TMF	Trial Master File
TMG	Trial Management Group
TSC	Trial Steering Committee
UAR	Unexpected Adverse Reaction
VFD	Ventilator Free Days
WHO	World Health Organisation

1. BACKGROUND AND DESIGN

The hypothesis under investigation is that in patients with moderate to severe ARDS, human umbilical cord derived CD362 enriched MSCs, (REALIST ORBCEL-C cells) are safe.

The aim of this study is to conduct a phase 1 clinical trial of human umbilical cord derived CD362 enriched MSCs, (REALIST ORBCEL-C cells), in patients with ARDS.

The primary objective is to assess the safety of a single intravenous infusion of REALIST ORBCEL-C cells in patients with ARDS.

The secondary objectives are in patients with moderate to severe ARDS to determine the effect of a single intravenous infusion of REALIST ORBCEL-C cells on:

1. Physiological indices of respiratory dysfunction reflecting severity of ARDS, as measured by oxygenation index (OI), respiratory compliance, and P/F ratio.
2. Sequential organ failure assessment (SOFA) score.
3. Alveolar and systemic markers of inflammatory responses.
4. Alveolar and systemic markers of cell specific injury.

The phase 1 trial is an open label dose escalation pilot study in which cohorts of subjects with moderate to severe ARDS will receive increasing doses of a single infusion of REALIST ORBCEL-C in a 3+3 design (Figure 1). We initially plan 3 cohorts with 3 subjects/cohort. Planned doses for the 3 cohorts pending absence of safety concerns are 100 x 10^6 cells, 200 x 10^6 cells and 400 x 10^6 cells. (Figure 2).

In PICO terms:

1. Population Young people (aged 16-17 years) and adult patients with moderate to severe ARDS
2. Intervention 100, 200 & 400x10^6 cell dose of REALIST ORBCEL-C
3. Comparator No comparator
4. Outcome Dose Limiting Toxicity

Figure 1: Flow diagram for the phase 1 trial

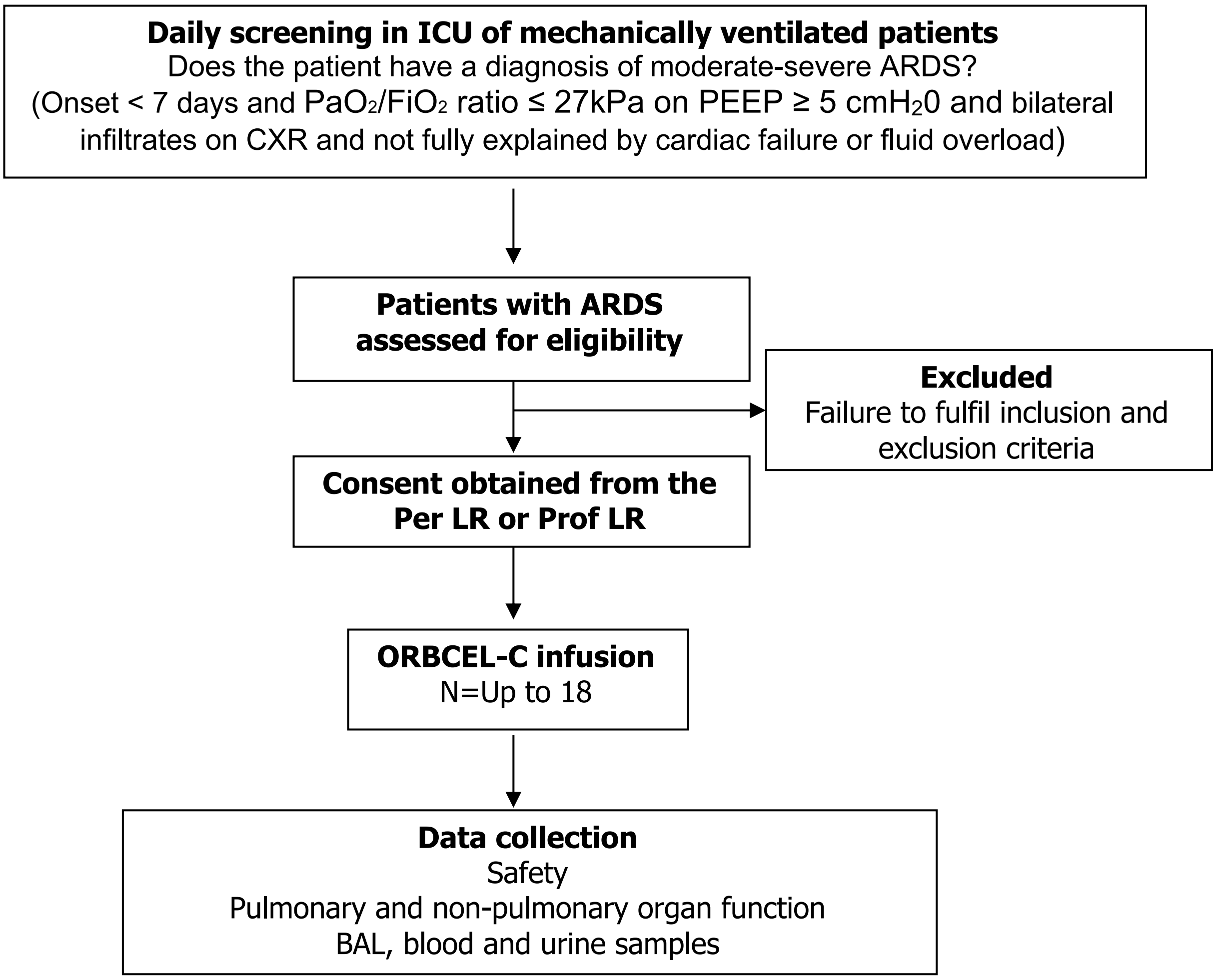

Figure 2: Flow diagram for the 3+3 trial design to determine the maximal tolerated dose (MTD)

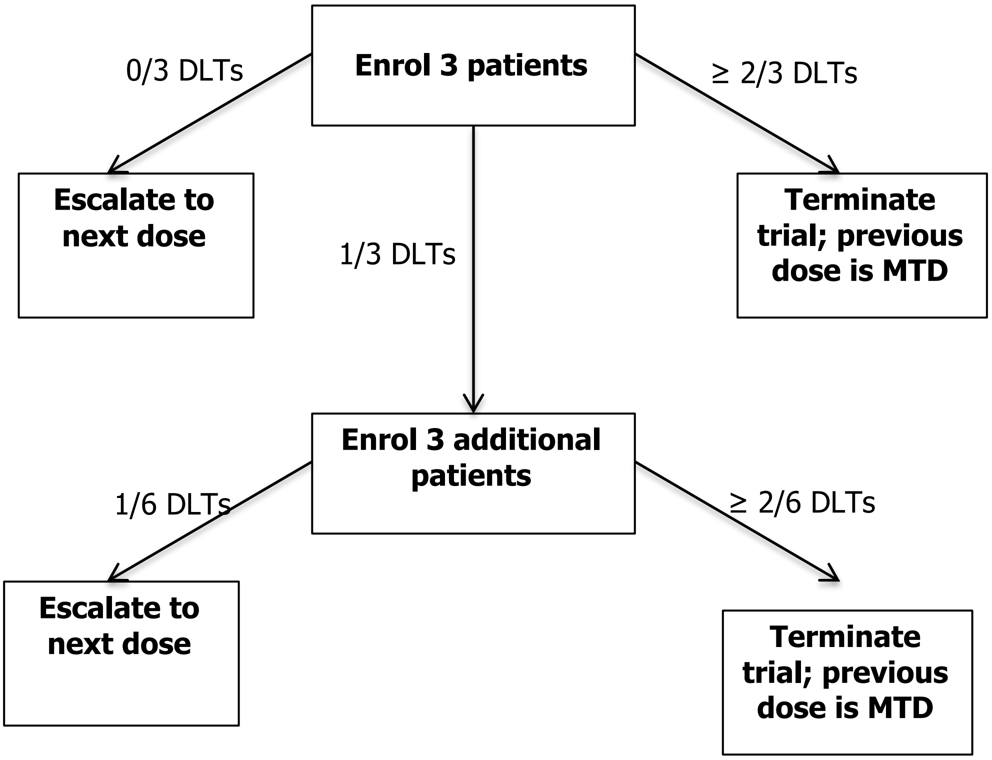

Patients will be assessed to day 7 for dose limiting toxicity (DLT) to determine dose escalation as described in the REALIST protocol and Dose Escalation Plan. The maximum dose will be 400 x 10^6 cells.

Full details of the background to the trial and its design are presented in the protocol.

2. OUTCOME MEASURES

2.1 Primary outcome measure(s)

The primary outcome of the phase 1 trial is the incidence of serious adverse events (SAEs).

The primary safety outcome is the incidence of serious adverse events (SAEs).

The primary efficacy outcome is oxygenation index (OI) at day 7.

OI is a physiological index of the severity of ARDS and measures both impaired oxygenation and the amount of mechanical ventilation delivered. OI is independently predictive of mortality in patients with ARDS. We have chosen day 7 as we expect this time interval will minimise the competing effects of death and extubation, while allowing a sufficient time interval for a biological effect to occur.

OI is calculated as (mean airway pressure (cm H_20) x FiO2 x 100) ÷ PaO_2 (kPa). These simple measurements are easily and routinely collected as part of standard ventilator practice.

2.2 Secondary outcome measures

The following secondary clinical outcomes will also be assessed:

1. OI at days 4 and 14.
2. Physiological indices of ARDS, as measured by respiratory compliance (Crs), driving pressure and P/F ratio on days 4, 7 and 14.
3. Organ failure as measured by the sequential organ failure assessment (SOFA) score on days 4, 7 and 14.

Outcomes will be measured at baseline and daily up to day 14 or until the patient is discharged from ICU or the patient dies.

Extubation, reintubation, ventilation free days at day 28, duration of ventilation, length of ICU and hospital stay as well as 28-day and 90-day mortality will be recorded. However, these important clinical outcomes are not included as outcome measures as the study is not adequately powered to assess these outcomes.

Patients will be followed up annually up to 2 years following recruitment.

2.3 Exploratory Outcome Measures

In order to determine the potential mechanism of action of MSCs the study will investigate the biological effect of MSCs on:

1. pulmonary and systemic inflammatory responses.
2. pulmonary and systemic indices of epithelial and endothelial function and injury.
3. Indices of coagulation.
4. Anti-HLA antibodies.
5. Cardiac function

3. DATA

3.1 CRF Forms and variables

Full details of the data to be collected and the timing of data collection are described in the trial protocol.

To ensure accurate, complete and reliable data are collected, the CTU will provide training to site staff in the format of investigator meetings and/or site initiation visits.

All data for an individual patient will be collected by the PI or designee and recorded in source documents/electronic CRF for the study. For routinely collected clinical data the NHS record will be the source document. Patient identification on the CRF will be through their unique participant study number, allocated at the time of recruitment. Data will be collected and recorded on the electronic CRF by the PI or designee as per the CRF submission guidelines.

If the participant is transferred to another hospital the PI or designated member of the site study team will liaise with the receiving hospital to ensure complete data capture as per CRF instruction. If this is not possible, the primary outcome must be collected as a minimum.

Data censorship for each trial participant will occur 90 days post recruitment.

3.2 Management of datasets

Following the entry of patient data into the study database, the data will be processed as per the CTU Standard Operating Procedures (SOPs) and the study specific Data Management Plan (DMP). Data queries will be generated electronically for site staff to clarify data or request missing information. The designated site staff will be required to respond to these queries. All queries will be responded to/resolved within the study database and amended in the study database.

At the time of analysis:

- The Data Manager in collaboration with the Study Statistician will extract data from MACRO following procedures as detailed in the SOP DM09 Database Closure/Lock and the corresponding study Data Management Plan (DMP).

3.3 Data completion schedule

All patients recruited to the Phase 1 and Phase 2 study must be evaluated according to the schedule of assessments as outlined in Table 3.3.1 Data will be collected at each of the following time points:

Table 3.3.1: Schedule of Assessments

	Day 0	Day 1	Day 2-3	Day 4	Day 5-6	Day 7	Day 8-13	Day 14	Day 15-28	Day 90 (+/- 14 days)	1 Year (+/- 30 days)	2 Year (+/- 30 days)
Eligibility assessment	X											
Informed consent	X											
Enrolment/ Randomisation	X											
Baseline data	X											
Daily data		X	X	X	X	X	X	X				
Chlorphenamine administration		X										
Study drug administration		X										
Adverse events		X	X	X	X	X	X	X	X	X		
ECHO data	X			X								
BAL sampling*	X			X								

Blood sampling[*,**]	X			X		X		X			X	X
Anti-HLA Ab[%]	X								X			
Urine sampling*	X			X		X		X				
Mortality[$]									X	X	X	X
Medical Event[#]											X	X

[*]Baseline BAL, blood samples and urine samples will be taken prior to study drug administration. Therefore this means they can be taken on day 0 or day 1.
[**]Blood for exploratory outcomes at year 1 and year 2 will be collected where possible
[%]Blood for anti-HLA Ab will be collected on day 0 and day 28 only
[$]Mortality, including cause of death.
[#]Any significant medical event

3.4 Data verification

The CTU will provide training to site staff on trial processes and procedures including CRF completion and data collection.

On-site monitoring visits during the trial will check the accuracy of entries on the electronic CRF against the source documents, the adherence to the protocol, procedures and Good Clinical Practice (GCP).

Quality control is implemented by the CTU in the form of Standard Operating Procedures (SOPs), which are defined to encompass aspects of the clinical data management process, and to ensure standardisation and adherence to International Conference of Harmonisation Good Clinical Practice (ICH-GCP) guidelines and regulatory requirements.

Data validation will be implemented and discrepancy reports will be generated following data entry to identify discrepancies such as out of range, inconsistencies or protocol deviations based on data validation checks programmed in the clinical trial database.

A Data Monitoring & Ethics Committee (DMEC) will be convened for the study to carry out reviews of the study data at staged intervals during the study.

3.5 Data coding

The variable codings will be as specified on the CRF.

4. DEFINITION OF TERMS

Give definition of any terms that require explanation.

E.g.

Term	Definition
Ventilator Free Days (VFDs)	VFDs to day 28 are defined as the number of days from the time of initiating unassisted breathing to day 28 after study drug administration, assuming survival for at least 48 hours after initiating unassisted breathing and continued unassisted breathing to day 28. If a patient returns to assisted breathing and subsequently achieves unassisted breathing to day 28, VFDs will be counted from the end of the last period of assisted breathing to day 28. A period of assisted breathing lasting less than 24 hours and for the purpose of a surgical procedure will not count against the VFD calculation. If a patient was receiving assisted breathing at day 27 or dies prior to day 28, VFDs will be zero. Patients transferred to another hospital or other health care facility will be followed to day 28 to assess this endpoint.
Discharge	Discharge from critical care is defined as first discharge to a ward in the hospital or another hospital; a transfer between ICUs is not considered a discharge from critical care. Hospital discharge is the first date that the patient is discharged to home/community, a transfer between hospitals is not considered as a hospital discharge
Time to extubation	Time to extubation will be counted from time of study drug administration to extubation.
Duration of Ventilation	Duration of ventilation will be counted from time of study drug administration to being successfully free from assisted breathing.
Oxygenation Index	OI is a physiological index of the severity of ARDS and measures both impaired oxygenation and the amount of mechanical ventilation delivered. OI is independently predictive of mortality in patients with ARDS [52, 53]. We have chosen day 7 as we expect this time interval will minimise the competing effects of death and extubation, while allowing a sufficient time interval for a biological effect to occur.<br><br>OI is calculated as (mean airway pressure (cm H_20) x FiO2 x 100) ÷ PaO_2 (kPa). These simple measurements are easily and routinely collected as part of standard ventilator practice.
Duration of stay	Duration of critical care and hospital stay will be counted from time of study drug administration to discharge.
Extubation	Extubation is defined as first time being successfully free from an endotracheal tube or a tracheostomy tube for 48hrs.
Unassisted breathing	Unassisted breathing i.e. no ventilatory support is defined as; extubated with supplemental oxygen or room air, or open T-tube breathing, or tracheostomy mask breathing, or CPAP without inspiratory pressure support for 48 hours. Patients receiving pressure support via non-invasive ventilation (except for sleep disordered breathing) or extra-corporeal lung support will be defined as receiving ventilatory support.

Driving Pressure	Plateau Pressure – PEEP
Dose Limiting Toxicity (DLT)	For the phase 1 trial, DLT is defined as any serious adverse reaction (SAR)

5. SAMPLE SIZE CALCULATIONS

The phase 1 trial will recruit up to 18 participants.

6. RANDOMISATION AND BLINDING

6.1 Randomisation

Participants will be allocated to receive either 100, 200 or 400 x 10^6 dose of REALIST ORBCEL-C.

After informed consent, patients will be allocated to the appropriate dose cohort via the CTU. Sites will be provided with trial specific allocation guidelines. Allocation will be completed by an appropriately trained and delegated member of the trial team. At the time of dose allocation, each patient will be allocated a unique Participant Study Number, which will be used throughout the study for participant identification. An entry will be recorded in the patients' medical notes noting enrolment into the study.

The CTU will manage the recruitment process to confirm when a contacting site can recruit a patient to a dose cohort to ensure only one patient across all sites is treated at a time and that each patient will receive treatment no less than 24 hours following the completion of treatment of another patient.

A dose escalation plan will be followed. Once the necessary number of patients have been recruited to a dose cohort the CTU will communicate to sites and not allow further patients to be allocated treatment until the DMEC has approved escalation to the next dose. An email will be sent to all site PIs when a dose cohort has been completed and when the DMEC has approved escalation to the next dose.

6.2 Blinding and Allocation Concealment

Allocation is unblinded for the Phase 1 trial.

7. ANALYSIS PRINCIPLES

For the Phase 1 trial no formal statistical analysis will be performed on safety data. The primary analysis will be descriptive and will focus on serious adverse events. The number of pre specified cell infusion associated events will also be reported. Descriptive analysis of pulmonary and non-pulmonary organ function will also be undertaken. We plan to publish the data from the phase 1 study.

The maximal tolerated dose up to 400x10^6cells will be proposed by the TMG and approved by the DMEC prior to use in the Phase 2 randomised controlled clinical trial.

A final analysis and report of the Phase 1 study is planned following the last patient's 90 day follow up.

All methodology issues for data analysis have been confirmed by the trial statistician from the Northern Ireland Clinical Trials Unit (NICTU).

Every effort will be made to minimise missing baseline and outcome data in this trial. The level and pattern of the missing data in the baseline variables and outcomes will be established by forming appropriate tables and the likely causes of any missing data will be investigated. This information will be used to determine whether the level and type of missing data has the potential to introduce bias into the analysis results for the proposed statistical methods, or substantially reduce the precision of estimates related to treatment effects. If necessary, these issues will be dealt with using multiple imputation or Bayesian methods for missing data as appropriate.

8. ANALYSIS DETAILS

The results of the analyses will be reported following the principles of the ICH E3 guidelines on the Structure and Content of Clinical Study Reports.

8.1 Recruitment and follow-up patterns

- Recruitment by year, centre.
- Withdrawals by site

8.2 CONSORT Flow Diagram – Dose 1 (100 x 10^6)

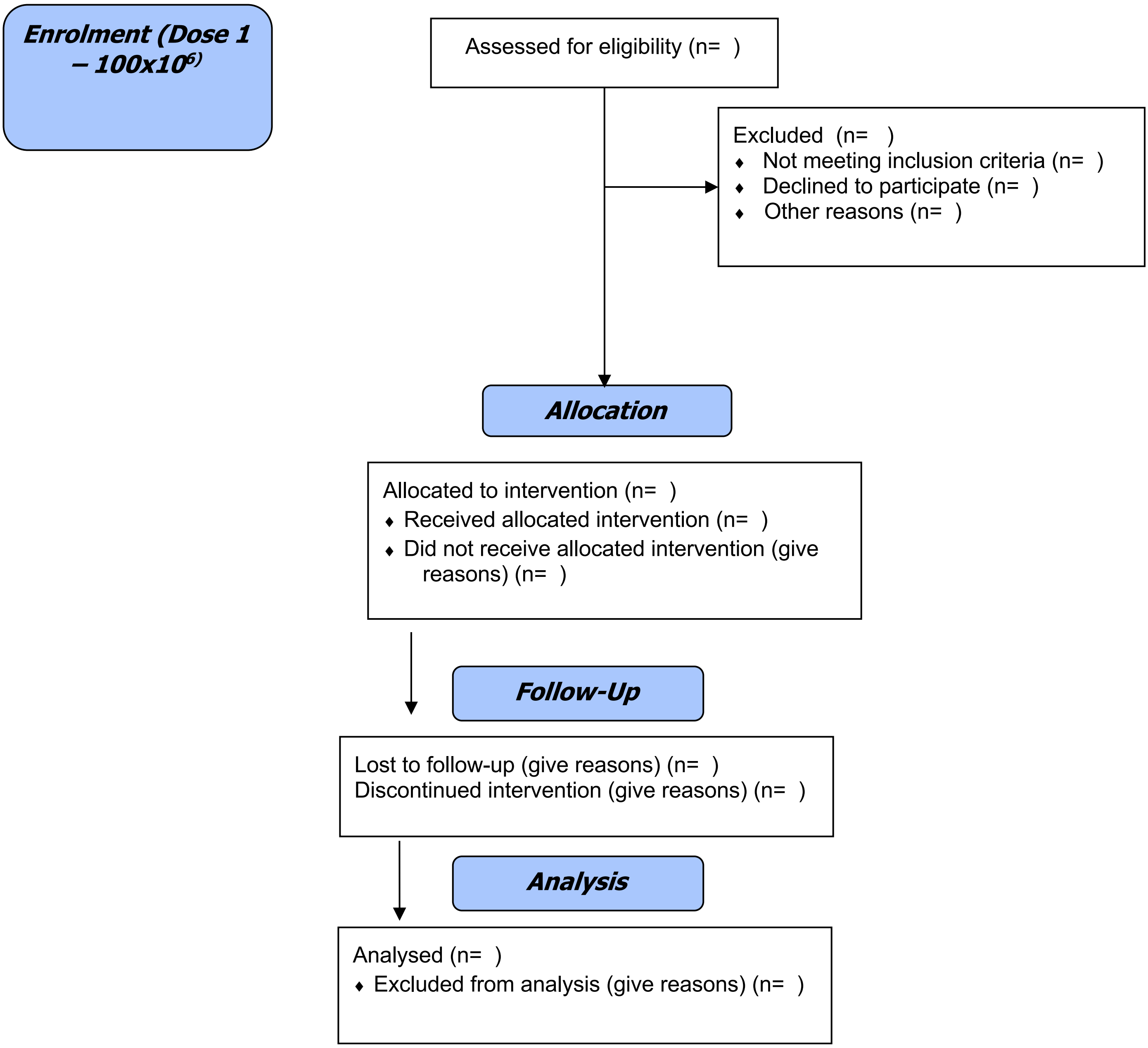

8.3 CONSORT Flow Diagram – Dose 2 (200 x 10^6)

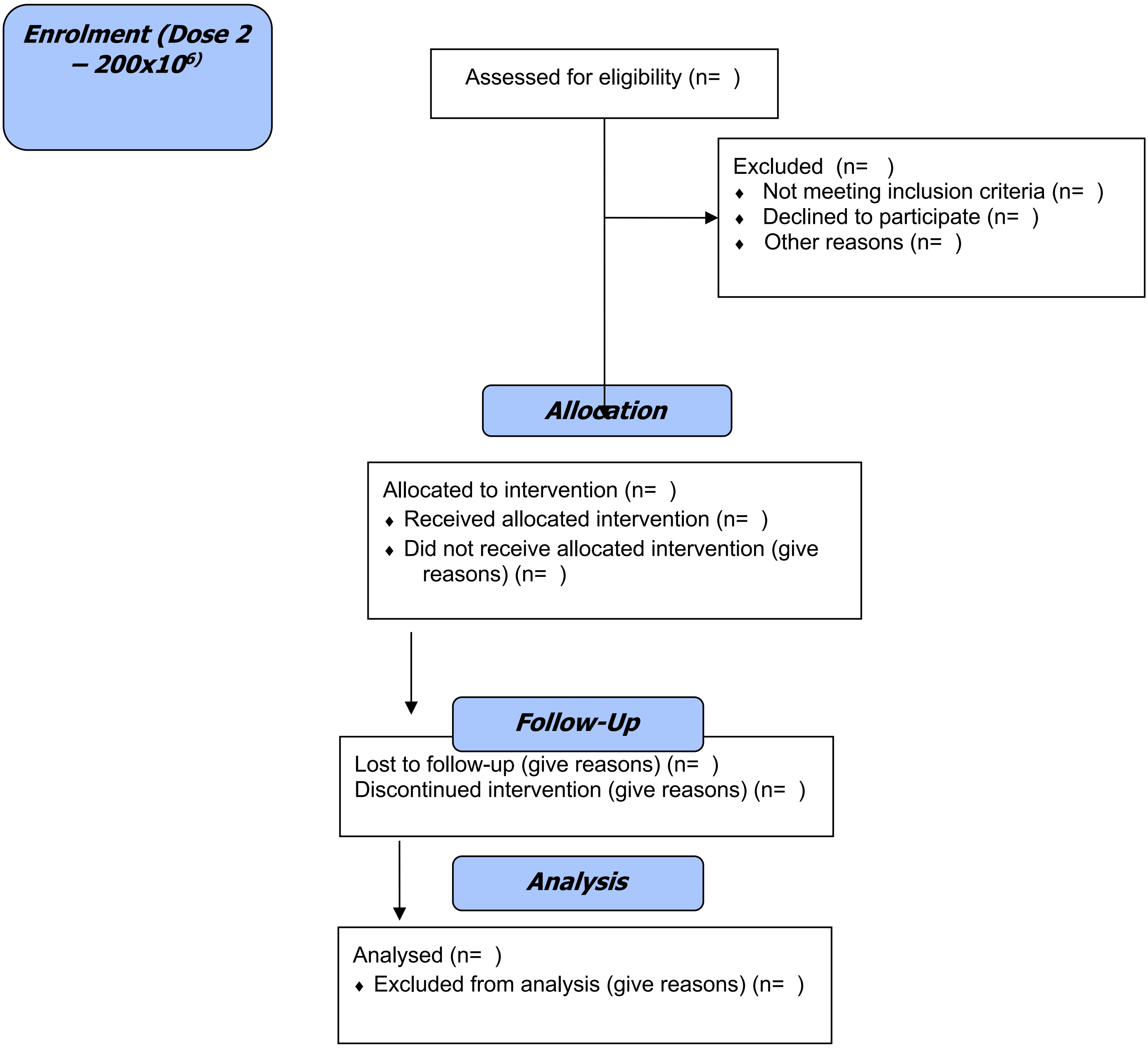

8.4 CONSORT Flow Diagram – Dose 3 (400 x 10^6)

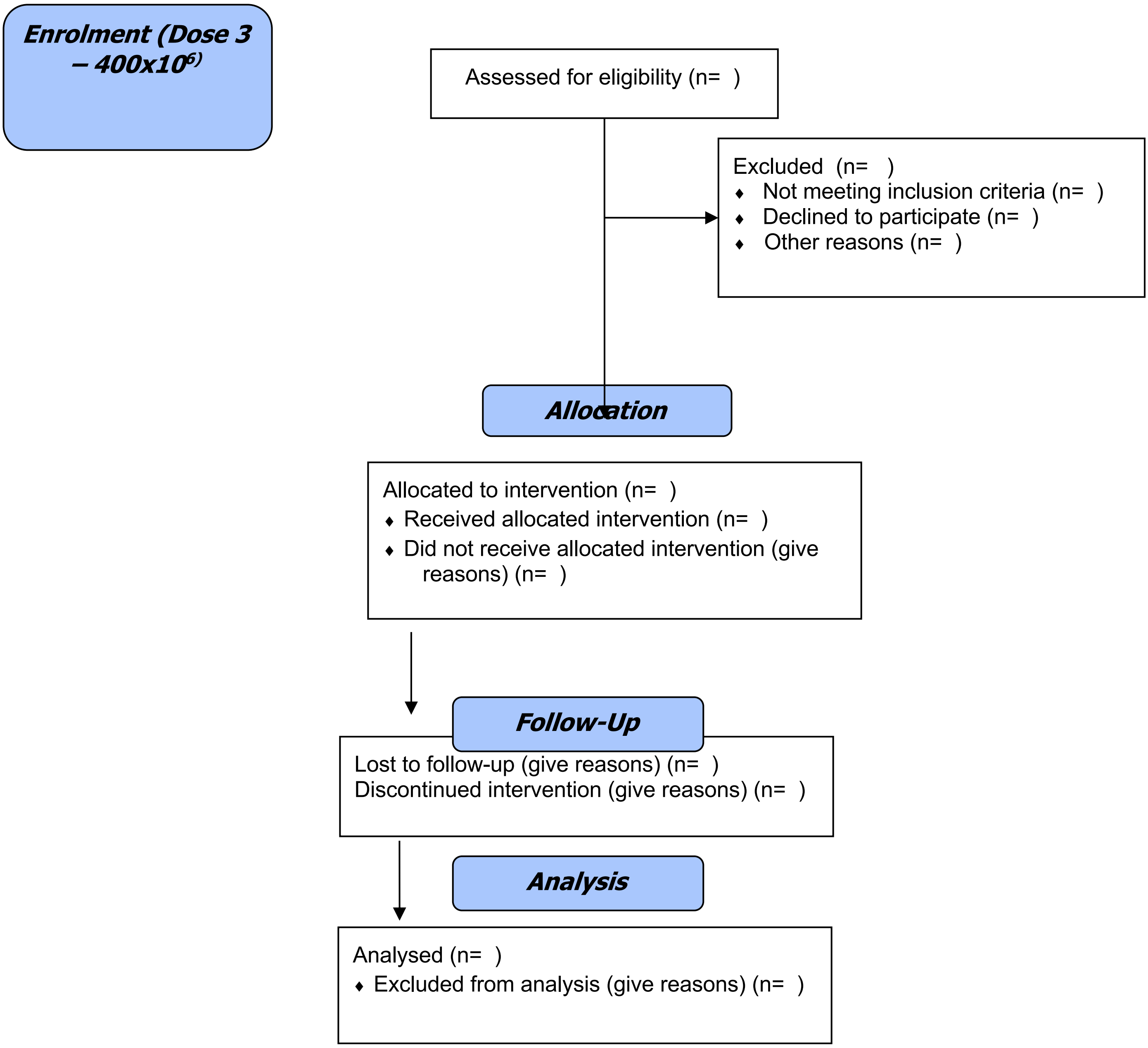

8.5 Safety

- AEs, SAEs, SARs and SUSARs, n(%) by dose

8.6 Baseline Characteristics

- Gender, n(%) by dose
- Age (years), mean(sd) by dose
- Temperature (°C) , mean(sd) by dose
- Aetiology of ARDS n(%) by dose
- APACHE II Score, mean(sd) by dose
- Murray Lung Injury Score (LIS) , mean(sd) by dose
- First Qualifying P/F Ratio, mean(sd) by dose
- Worst PaO2/FiO2 ratio (Day 0/24 hrs prior to randomisation) [Min, Max] , mean(sd) by dose
- Total SOFA Score, mean(sd) by dose
- Oxygenation Index, mean(sd) by dose
- Lowest Mean Arterial Pressure (mmHg) , mean(sd) by dose
- PEEP (cmH2O) , mean(sd) by dose
- Plateau Pressure (cmH2O) , mean(sd) by dose
- Driving Pressure (cmH2O), mean(sd) by dose
- Mode of Ventilation, n(%) by dose
- Tidal Volume (Vt) ml/kg PBW, mean(sd) by dose
-

8.7 Trial treatment

- Study drug given, n(%) by dose
- Full dose given, n(%) by dose
- Reasons for termination of study drug, n(%) by dose
- Protocol violations , n(%) by dose
- Post-randomisation withdrawal, n(%) by dose
- Ineligible patient, n(%) by dose
- Did not receive allocated treatment, n(%) by dose

8.8 Trial Outcomes

- Primary safety outcome; incidence of SAEs, n(%) by dose
- Oxygenation Index (OI) at day 4, day 7 and day 14, mean(sd) by dose
- Respiratory compliance and P/F ratio at day 4, day 7 and day 14, mean(sd) by dose
- Sequential Organ Failure Assessment (SOFA) score at day 4, day 7 and day 14, mean(sd) by dose
- Time to 1st successful Extubation, mean(sd) by dose and median(IQR) by dose.
- Total number of reintubations after a planned extubation, mean(sd) by dose
- Average number of reintubations per patient, mean(sd) by dose
- Ventilation Free Days at day 28, mean(sd) by dose
- Duration of Ventilation, mean(sd) by dose
- Length of ICU stay , mean(sd) by dose
- Length of hospital stay , mean(sd) by dose
- 28 day mortality , n(%) by dose
- 90 day mortality, n(%) by dose

8.9 Main Clinical Lab Assessments

- AST, mean(sd) by dose
- ALT, mean(sd) by dose
- ALP, mean(sd) by dose
- CRP, mean(sd) by dose
- PT, mean(sd) by dose
- APTT, mean(sd) by dose
- Fibrinogen, mean(sd) by dose
- Hb, mean(sd) by dose
- WBC, mean(sd) by dose
- Neutrophils, mean(sd) by dose
- Lowest eGFR, mean(sd) by dose
- Highest Urea, mean(sd) by dose

9. ADDITIONAL INFORMATION

9.1 Trial Steering Committee (TSC)

The conduct of the trial will be overseen by a Trial Steering Committee (TSC) on behalf of the Sponsor/Funder. The TSC will include the Chief Investigator (CI), 2 of the co-investigators and a group of experienced critical care clinicians and trialists as well as a "lay" representative. Annual meetings will be held, however as the Data Monitoring and Ethics Committee (DMEC) will meet during the phase 1 and 2 trial, the TSC may be convened to discuss issues and recommendations raised by the DMEC, in addition to the scheduled annual meetings. The roles and responsibilities of the TSC will be detailed in the Trial Steering Committee Charter. The TSC, in the development of this protocol and throughout the trial, will take responsibility for monitoring and guiding overall progress, scientific standards, operational delivery and protecting the rights and safety of trial participants. Meetings will be formally minuted and stored in the Trial Master File (TMF).

9.2 Data Monitoring and Ethics Committee (DMEC)

A Data Monitoring and Ethics Committee (DMEC) will be appointed comprising two clinicians with experience in undertaking clinical trials / caring for critically ill patients / cell therapy and a statistician who are independent of the trial. The DMEC will meet to agree conduct and remit, and the roles and responsibilities of the DMEC will be detailed in the Data Monitoring and Ethics Committee Charter. The DMEC will be convened after each dose cohort of patients have been enrolled into the phase 1 trial and completed at least 7 days follow-up to approve dose escalation. When 7 days of follow-up data for all study subjects in the phase 1 study are available the TMG will review the data and propose a cell dose for the phase 2 trial. This recommendation will be submitted to the DMEC for approval prior to initiating the phase 2 trial. In the phase 2 trial the DMEC will be convened after 20, 40 and 60 patients have been recruited and completed at least 7 days follow up. In the event of any safety concerns additional unplanned DMEC meetings will be convened. The DMEC's responsibility is to safeguard the interests of the trial participants, in particular with regard to safety, and assist and advise the TSC so as to protect the validity and credibility of the trial. The DMEC will monitor recruitment, adverse events and outcome data. During the recruitment period, reports will be provided to

the DMEC which will include information on recruitment, AEs reported, and deaths from all causes at 28 days, along with any other data that the committee may request. As this is a phase 2 trial, an interim analysis of efficacy is not planned although this issue can be discussed by the DMEC as required. Meetings will be formally minuted and stored in the Trial Master File (TMF).

Following a recommendation from the DMEC, the TSC will decide what actions, if any, are required. It will be the responsibility of the TSC to inform the Sponsor if concerns exist about patient safety, following which the Sponsor will take appropriate action.

10. SIGNATURES OF APPROVAL

Date: 08/07/2019
Version: 1.0 Final
This document has completed a final review and is understood and approved by the following:

Danny McAuley		
Chief Investigator Name	*Chief Investigator Signature*	Date dd/mm/yyyy
Clíona McDowell		
Senior Statistician or designee Name	*Senior Statistician or designee Signature*	Date dd/mm/yyyy
Cliona McDowell		
Study Statistician Name	*Study Statistician Signature*	Date dd/mm/yyyy

APPENDIX 1: EXAMPLE DRAFT SUMMARY TABLES

Table x.x.x. Baseline Characteristics at trial entry

		Dose 1	Dose 2	Dose 3
		n = <n>	n = <n>	n = <n>
Gender	Male<br>Female	n(%)<br>n(%)	n(%)<br>n(%)	n(%)<br>n(%)
Age (years)		xx.x (xx.x)	xx.x (xx.x)	xx.x (xx.x)
Temperature (°C)		xx.x (xx.x)	xx.x (xx.x)	xx.x (xx.x)
Aetiology of ARDS	Smoke/toxin inhalation	n (xx.x%)	n (xx.x%)	n (xx.x%)
	Gastric content aspiration	n (xx.x%)	n (xx.x%)	n (xx.x%)
	Near drowning	n (xx.x%)	n (xx.x%)	n (xx.x%)
	Thoracic trauma	n (xx.x%)	n (xx.x%)	n (xx.x%)
	Pneumonia	n (xx.x%)	n (xx.x%)	n (xx.x%)
	Sepsis	n (xx.x%)	n (xx.x%)	n (xx.x%)
	Cardiopulmonary bypass	n (xx.x%)	n (xx.x%)	n (xx.x%)
	Pancreatitis	n (xx.x%)	n (xx.x%)	n (xx.x%)
	Non-thoracic trauma	n (xx.x%)	n (xx.x%)	n (xx.x%)
	Other	n (xx.x%)	n (xx.x%)	n (xx.x%)
APACHE II Score		xx.x (xx.x)	xx.x (xx.x)	xx.x (xx.x)
Murray Lung Injury Score (LIS)		xx.x (xx.x)	xx.x (xx.x)	xx.x (xx.x)
First Qualifying P/F Ratio		xx.x (xx.x)	xx.x (xx.x)	xx.x (xx.x)
Worst PaO_2/FiO_2 ratio (Day 0/24 hrs prior to randomisation) [Min, Max]		xx.x (xx.x)	xx.x (xx.x)	xx.x (xx.x)
Total SOFA Score		xx.x (xx.x)	xx.x (xx.x)	xx.x (xx.x)
Oxygenation Index		xx.x (xx.x)	xx.x (xx.x)	xx.x (xx.x)
Lowest Mean Arterial Pressure (mmHg)		xx.x (xx.x)	xx.x (xx.x)	xx.x (xx.x)
PEEP (cmH_2O)		xx.x (xx.x)	xx.x (xx.x)	xx.x (xx.x)
Plateau Pressure (cmH_2O)		xx.x (xx.x)	xx.x (xx.x)	xx.x (xx.x)
Driving Pressure (cmH_2O)		xx.x (xx.x)	xx.x (xx.x)	xx.x (xx.x)
Mode of Ventilation	SIMV	n (xx.x%)	n (xx.x%)	n (xx.x%)
	PS	n (xx.x%)	n (xx.x%)	n (xx.x%)
	Other	n (xx.x%)	n (xx.x%)	n (xx.x%)
	None	n (xx.x%)	n (xx.x%)	n (xx.x%)
Tidal Volume (Vt) ml/kg PBW [Min, Max]		xx.x (xx.x)	xx.x (xx.x)	xx.x (xx.x)

Mean (SD) presented for continuous variables and no. (%) for all categorical variables.

Table x.x.x. Treatment after Trial Entry

	Dose 1	Dose 2	Dose 3	Total
	n = <n>	n = <n>	n = <n>	n = <n>
Study drug given	n(%)	n(%)	n(%)	n(%)
Full dose given	n(%)	n(%)	n(%)	n(%)
Reasons for termination of study drug<br>Study drug related adverse event<br>Study drug expiry<br>Death or discontinuation of active treatment<br>Request from PerLR or ProLR to withdraw the patient from the study<br>Decision by the attending clinician on safety grounds.	 n(%)<br>n(%)<br>n(%)<br>n(%)<br>n(%)	 n(%)<br>n(%)<br>n(%)<br>n(%)<br>n(%)	 n(%)<br>n(%)<br>n(%)<br>n(%)<br>n(%)	 n(%)<br>n(%)<br>n(%)<br>n(%)<br>n(%)
Protocol violations:<br>Eligibility<br>Study Drug Administration<br>SAE reporting<br>Other<br><br>Post-randomisation withdrawal<br>Refused use of data already collected<br>Refused data collection from NHS records<br>Withdrew from follow-up<br><br>Did not receive allocated treatment	 n(%)<br>n(%)<br>n(%)<br>n(%)<br><br><br>n(%)<br>n(%)<br>n(%)<br><br>n(%)	 n(%)<br>n(%)<br>n(%)<br>n(%)<br><br><br>n(%)<br>n(%)<br>n(%)<br><br>n(%)	 n(%)<br>n(%)<br>n(%)<br>n(%)<br><br><br>n(%)<br>n(%)<br>n(%)<br><br>n(%)	 n(%)<br>n(%)<br>n(%)<br>n(%)<br><br><br>n(%)<br>n(%)<br>n(%)<br><br>n(%)

Table x.x.x Main Clinical Outcome variables

	**Dose 1**	**Dose 2**	**Dose 3**	**Total**
Primary safety outcome; incidence of SAEs	n(%)	n(%)	n(%)	n(%)
Oxygenation Index (OI) at;<br>Day 4<br>Day 7<br>Day 14	xx.x (xx.x)<br>xx.x (xx.x)<br>xx.x (xx.x)	xx.x (xx.x)<br>xx.x (xx.x)<br>xx.x (xx.x)	xx.x (xx.x)<br>xx.x (xx.x)<br>xx.x (xx.x)	xx.x (xx.x)<br>xx.x (xx.x)<br>xx.x (xx.x)
Respiratory compliance and P/F ratio at;<br>Day 4<br>Day 7<br>Day 14	xx.x (xx.x)<br>xx.x (xx.x)<br>xx.x (xx.x)	xx.x (xx.x)<br>xx.x (xx.x)<br>xx.x (xx.x)	xx.x (xx.x)<br>xx.x (xx.x)<br>xx.x (xx.x)	xx.x (xx.x)<br>xx.x (xx.x)<br>xx.x (xx.x)
Sequential Organ Failure Assessment (SOFA) score at;<br>Day 4<br>Day 7<br>Day 14	xx.x (xx.x)<br>xx.x (xx.x)<br>xx.x (xx.x)	xx.x (xx.x)<br>xx.x (xx.x)<br>xx.x (xx.x)	xx.x (xx.x)<br>xx.x (xx.x)<br>xx.x (xx.x)	xx.x (xx.x)<br>xx.x (xx.x)<br>xx.x (xx.x)
Additional Outcomes				
Time to 1st successful extubation (hours)	xx.x (xx.x)<br>x(x,x)	xx.x (xx.x)<br>x(x,x)	xx.x (xx.x)<br>x(x,x)	xx.x (xx.x)<br>x(x,x)
Total number of reintubation after a planned extubation	xx.x (xx.x)	xx.x (xx.x)	xx.x (xx.x)	xx.x (xx.x)
Average number of reintubations per patient	xx.x (xx.x)	xx.x (xx.x)	xx.x (xx.x)	xx.x (xx.x)
Ventilation Free Days at day 28	xx.x (xx.x)	xx.x (xx.x)	xx.x (xx.x)	xx.x (xx.x)
Duration of Ventilation	xx.x (xx.x)	xx.x (xx.x)	xx.x (xx.x)	xx.x (xx.x)
Length of ICU stay	xx.x (xx.x)	xx.x (xx.x)	xx.x (xx.x)	xx.x (xx.x)
Length of hospital stay	xx.x (xx.x)	xx.x (xx.x)	xx.x (xx.x)	xx.x (xx.x)
28 day mortality	n(%)	n(%)	n(%)	n(%)
90 day mortality	n(%)	n(%)	n(%)	n(%)

Mean (SD), median(IQR) or n(%) presented

Table x.x.x Main Clinical Lab Assessments

	**Dose 1**	**Dose 2**	**Dose 3**	**Total**
AST				
Day 0	xx.x (xx.x)	xx.x (xx.x)	xx.x (xx.x)	xx.x (xx.x)
Day 1	xx.x (xx.x)	xx.x (xx.x)	xx.x (xx.x)	xx.x (xx.x)
Day 2 etc…	xx.x (xx.x)	xx.x (xx.x)	xx.x (xx.x)	xx.x (xx.x)
ALT				
Day 0	xx.x (xx.x)	xx.x (xx.x)	xx.x (xx.x)	xx.x (xx.x)
Day 1	xx.x (xx.x)	xx.x (xx.x)	xx.x (xx.x)	xx.x (xx.x)
Day 2 etc…	xx.x (xx.x)	xx.x (xx.x)	xx.x (xx.x)	xx.x (xx.x)
ALP				
Day 0	xx.x (xx.x)	xx.x (xx.x)	xx.x (xx.x)	xx.x (xx.x)
Day 1	xx.x (xx.x)	xx.x (xx.x)	xx.x (xx.x)	xx.x (xx.x)
Day 2 etc…	xx.x (xx.x)	xx.x (xx.x)	xx.x (xx.x)	xx.x (xx.x)
CRP				
Day 0	xx.x (xx.x)	xx.x (xx.x)	xx.x (xx.x)	xx.x (xx.x)
Day 1	xx.x (xx.x)	xx.x (xx.x)	xx.x (xx.x)	xx.x (xx.x)
Day 2 etc…	xx.x (xx.x)	xx.x (xx.x)	xx.x (xx.x)	xx.x (xx.x)
PT				
Day 0	xx.x (xx.x)	xx.x (xx.x)	xx.x (xx.x)	xx.x (xx.x)

Day 1	xx.x (xx.x)	xx.x (xx.x)	xx.x (xx.x)	xx.x (xx.x)
Day 2 etc…	xx.x (xx.x)	xx.x (xx.x)	xx.x (xx.x)	xx.x (xx.x)
APTT				
Day 0	xx.x (xx.x)	xx.x (xx.x)	xx.x (xx.x)	xx.x (xx.x)
Day 1	xx.x (xx.x)	xx.x (xx.x)	xx.x (xx.x)	xx.x (xx.x)
Day 2 etc…	xx.x (xx.x)	xx.x (xx.x)	xx.x (xx.x)	xx.x (xx.x)
Fibrinogen				
Day 0	xx.x (xx.x)	xx.x (xx.x)	xx.x (xx.x)	xx.x (xx.x)
Day 1	xx.x (xx.x)	xx.x (xx.x)	xx.x (xx.x)	xx.x (xx.x)
Day 2 etc…	xx.x (xx.x)	xx.x (xx.x)	xx.x (xx.x)	xx.x (xx.x)
Hb				
Day 0	xx.x (xx.x)	xx.x (xx.x)	xx.x (xx.x)	xx.x (xx.x)
Day 1	xx.x (xx.x)	xx.x (xx.x)	xx.x (xx.x)	xx.x (xx.x)
Day 2 etc…	xx.x (xx.x)	xx.x (xx.x)	xx.x (xx.x)	xx.x (xx.x)
WBC				
Day 0	xx.x (xx.x)	xx.x (xx.x)	xx.x (xx.x)	xx.x (xx.x)
Day 1	xx.x (xx.x)	xx.x (xx.x)	xx.x (xx.x)	xx.x (xx.x)
Day 2 etc…	xx.x (xx.x)	xx.x (xx.x)	xx.x (xx.x)	xx.x (xx.x)
Neutrophils				
Day 0	xx.x (xx.x)	xx.x (xx.x)	xx.x (xx.x)	xx.x (xx.x)
Day 1	xx.x (xx.x)	xx.x (xx.x)	xx.x (xx.x)	xx.x (xx.x)
Day 2 etc…	xx.x (xx.x)	xx.x (xx.x)	xx.x (xx.x)	xx.x (xx.x)
Lowest eGFR				
Day 0	xx.x (xx.x)	xx.x (xx.x)	xx.x (xx.x)	xx.x (xx.x)
Day 1	xx.x (xx.x)	xx.x (xx.x)	xx.x (xx.x)	xx.x (xx.x)
Day 2 etc…	xx.x (xx.x)	xx.x (xx.x)	xx.x (xx.x)	xx.x (xx.x)
Highest Urea				
Day 0	xx.x (xx.x)	xx.x (xx.x)	xx.x (xx.x)	xx.x (xx.x)
Day 1	xx.x (xx.x)	xx.x (xx.x)	xx.x (xx.x)	xx.x (xx.x)
Day 2 etc…	xx.x (xx.x)	xx.x (xx.x)	xx.x (xx.x)	xx.x (xx.x)

Table x.x.x. Safety by Treatment Group

		Dose 1		Dose 2		Dose 3		Total	
		No. Events	No. Patients	No. Events	No. Patients	No. Events	No. Patients	No. Events	No. Patients
AEs, SAEs and SUSARs	Total AEs	n(%)	n(%)	n(%)	n(%)	n(%)	n(%)	n(%)	n(%)
	Related to study drug (AR)	n(%)	n(%)	n(%)	n(%)	n(%)	n(%)	n(%)	n(%)
	Total SAEs	n(%)	n(%)	n(%)	n(%)	n(%)	n(%)	n(%)	n(%)
	Related to study drug (SAR) Dose limiting toxicity is defined as any SAR.	n(%)	n(%)	n(%)	n(%)	n(%)	n(%)	n(%)	n(%)
	Related to study drug and unexpected (SUSAR)	n(%)	n(%)	n(%)	n(%)	n(%)	n(%)	n(%)	n(%)
SAEs	Cardiac Arrhythmia	n(%)	n(%)	n(%)	n(%)	n(%)	n(%)	n(%)	n(%)
	Cardiac General	n(%)	n(%)	n(%)	n(%)	n(%)	n(%)	n(%)	n(%)
	Gastrointestinal	n(%)	n(%)	n(%)	n(%)	n(%)	n(%)	n(%)	n(%)
	Etc…..	n(%)	n(%)	n(%)	n(%)	n(%)	n(%)	n(%)	n(%)
AEs	Cardiac Arrhythmia	n(%)	n(%)	n(%)	n(%)	n(%)	n(%)	n(%)	n(%)
	Cardiac General	n(%)	n(%)	n(%)	n(%)	n(%)	n(%)	n(%)	n(%)
	Gastrointestinal	n(%)	n(%)	n(%)	n(%)	n(%)	n(%)	n(%)	n(%)
	Etc…..	n(%)	n(%)	n(%)	n(%)	n(%)	n(%)	n(%)	n(%)
