# Supplementary material for: Repair of acute respiratory distress syndrome by stromal cell administration (REALIST) trial: A phase 1 trial
Source: eClinicalMedicine. 2021 Oct 24;41:101167. doi: 10.1016/j.eclinm.2021.101167 (PMC8551601; doi:10.1016/j.eclinm.2021.101167)
Supplement: Supplementary file 8 [file mmc8.docx]

Captions for supplementary material

Supplemental Table 1: Primary and secondary outcome variables

Supplemental Table 2: Biomarker studies at day 0, 4, 7 and 14

Supplemental Table 3: Routine clinical laboratory measurements daily to day 14

Supplemental Table 4: Anti-HLA antibody response at day 0 and day 28

Supplemental Figure 1: Pulmonary and non-pulmonary organ function outcomes using imputed data.

Supplemental file 1: Protocol v 3.0 26.06.2019

Supplemental file 2: Phase 1 Statistical Analysis Plan
