# Supplementary material for: Repair of acute respiratory distress syndrome by stromal cell administration (REALIST) trial: A phase 1 trial
Source: eClinicalMedicine. 2021 Oct 24;41:101167. doi: 10.1016/j.eclinm.2021.101167 (PMC8551601; doi:10.1016/j.eclinm.2021.101167)

Supplemental Figure 1: Pulmonary (Oxygenation Index, P/F ratio, Respiratory Compliance, Driving pressure) and non-pulmonary organ function (SOFA score) outcomes using imputed data.

Mean (SD) are provided for each dose cohort (dose 1: 100 x10^6; dose 2: 200 x 10^6; dose 3: 400 x 10^6) at day 0 (baseline), day 4, day 7 and day 14. Imputed values are derived from last available observed value.
A) Oxygenation index (OI) B) P/F Ratio C) Respiratory Compliance D) Driving Pressure E) SOFA Score

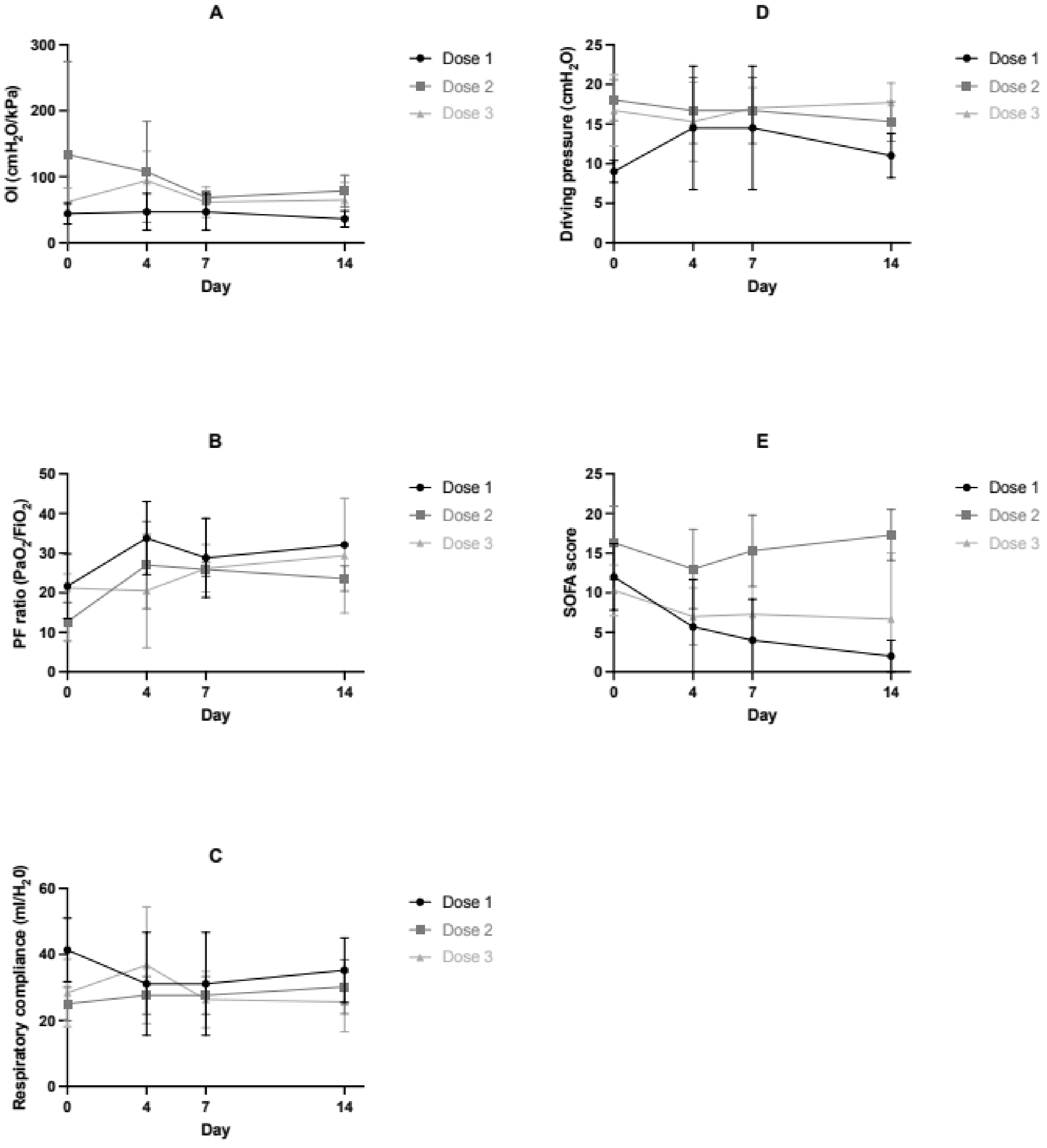

Supplement: Supplementary file 8 [file mmc7.docx]
